# Structure and Growth Control of Organic–Inorganic Halide Perovskites for Optoelectronics: From Polycrystalline Films to Single Crystals

**DOI:** 10.1002/advs.201500392

**Published:** 2016-03-15

**Authors:** Yani Chen, Minhong He, Jiajun Peng, Yong Sun, Ziqi Liang

**Affiliations:** ^1^ Department of Materials ScienceFudan UniversityShanghai200433P.R. China

**Keywords:** perovskites, film formation, single crystals, crystal growth, optoelectronics

## Abstract

Recently, organic–inorganic halide perovskites have sparked tremendous research interest because of their ground‐breaking photovoltaic performance. The crystallization process and crystal shape of perovskites have striking impacts on their optoelectronic properties. Polycrystalline films and single crystals are two main forms of perovskites. Currently, perovskite thin films have been under intensive investigation while studies of perovskite single crystals are just in their infancy. This review article is concentrated upon the control of perovskite structures and growth, which are intimately correlated for improvements of not only solar cells but also light‐emitting diodes, lasers, and photodetectors. We begin with the survey of the film formation process of perovskites including deposition methods and morphological optimization avenues. Strategies such as the use of additives, thermal annealing, solvent annealing, atmospheric control, and solvent engineering have been successfully employed to yield high‐quality perovskite films. Next, we turn to summarize the shape evolution of perovskites single crystals from three‐dimensional large sized single crystals, two‐dimensional nanoplates, one‐dimensional nanowires, to zero‐dimensional quantum dots. Siginificant functions of perovskites single crystals are highlighted, which benefit fundamental studies of intrinsic photophysics. Then, the growth mechanisms of the previously mentioned perovskite crystals are unveiled. Lastly, perspectives for structure and growth control of perovskites are outlined towards high‐performance (opto)electronic devices.

## 1 Introduction

The last few years have witnessed the unprecedented rapid development of a new class of solar cells based on organic–inorganic halide perovskites.^1^, ^2^, ^3^, ^4^ Initial studies of organometal trihalides as sensitizers for liquid‐electrolyte‐based dye‐sensitized solar cells (DSSCs) achieved a high photovoltage close to 1.0 V and a power conversion efficiency (PCE) of ≈3% in 2009.^5^ In late 2012, Park et al. fabricated a solid‐state mesoscopic perovskite‐sensitized solar cell and boosted the PCE up to 9.7%.^6^ The breakthrough in perovskite photovoltaics (PVs) began with Snaith and co‐workers' report, which employed mesoporous alumina scaffold and methylammonium lead iodide chloride to fabricate meso‐superstructured solar cells (MSSCs), resulting in 10.9% efficiency.^7^ Later, the same group further developed planar heterojunction (PHJ) perovskite solar cells, which removed the mesoporous layer and exhibited a PCE of 15.4%.^8^ Research on organometal halide perovskites based solar cells have since been undertaken drastically and skyrocketed a certified PCE value of 21.0%, which largely exceeded those of organic photovoltaics (OPVs), DSSCs and rivaled those of conventional thin‐film PVs such as crystalline silicon (c‐Si), gallium arsenide (GaAs) and copper indium gallium (di)selenide (CIGS) cells.^9^ Apart from their extraordinary photovoltaic properties, organometal halide perovskites are excellent candidates for realization of other electronic applications such as lasers, photodetectors, and light‐emitting diodes (LEDs).^10^, ^11^, ^12^, ^13^, ^14^, ^15^, ^16^

**Figure 1:**
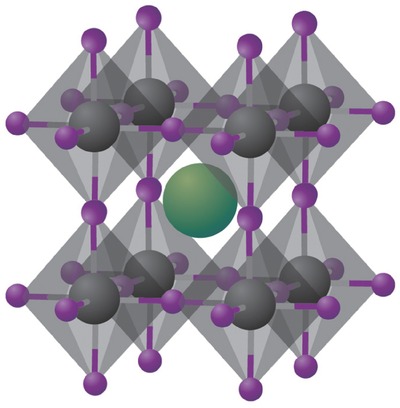
Crystal structure of cubic metal halide perovskites with the generic chemical formula *ABX*_3_. Organic or inorganic cations occupy position *A* (green) whereas metal cations and halides occupy the *B* (grey) and *X* (purple) positions, respectively. Reproduced with permission.^17^ Copyright 2014, Nature Publishing Group.

Perovskites, named after Russian mineralogist L. A. Perovski, initially referred to a calcium titanium oxide mineral composed of calcium titanate (CaTiO_3_). Such terminology has since been extended to the kind of compounds that take the similar crystal structure as CaTiO_3_. The class of hybrid organic−inorganic perovskites adopts the general perovskite chemical formula AB*X*_3_, where A is monovalent organic cation—typically CH_3_NH_3_^+^ (i.e., MA^+^) and HC(NH_2_)_2_^+^ (i.e., FA^+^), B is metal cation (i.e., Pb^2+^, Sn^2+^), and *X* is halide anion (i.e., Cl^−^, Br^−^, I^−^ or their mixtures). Different than conventional perovskites, the organic part A serves as structural template and affords solution processability. The most commonly studied hybrid perovskites include methylammonium lead triiodide perovskite (CH_3_NH_3_PbI_3_), mixed halide perovskite (CH_3_NH_3_PbI_3‐*x*_Cl*_x_* and CH_3_NH_3_PbI_3‐*x*_Br*_x_*) and formamidinium lead triiodide (NH_2_CHNH_2_PbI_3_, FAPbI_3_). In a typical perovskite crystal structure, B occupies the center of an octahedral [B*X*_6_]^4−^ cluster, while A is 12‐fold cuboctahedral coordinated with X anions, as shown in **Figure** **1**.^17^, ^18^ The formability of this crystal structure can be estimated by Goldschmidt tolerance factor (*t*), which is displayed in Equation (1).^19^

(1) $$t=\frac{r_{A}+r_{X}}{\sqrt{2}\left(r_{B}+r_{X}\right)}$$

Where *r_A_*, *r_B_*, and *r_X_* are the effective ionic radii for *A*, *B*, and *X* ions, respectively. Another factor, called octahedral factor (*μ*), *μ* = *r*_B_/*r_X_*, is also used to evaluate stability of perovskite. It is believed that perovskites can be stabilized when *t* lies in the range of 0.813−1.107 and *μ* is in the range of 0.442−0.895.^20^ Such unique structure renders perovskite with a host of intriguing characteristics such as high absorption coefficient,^6^ wide absorption range, tunable bandgaps,^21^ low exciton binding energy,^22^ long electron and hole diffusion lengths,^23^, ^24^ high ambipolar charge mobilities,^25^, ^26^ and extended charge carrier lifetime.^27^

Polycrystalline films and single crystals are two main forms of perovskites. For the film format, a fine control of perovskite crystallization is critical to film morphology such as uniformity and surface coverage, which essentially determine the performance of solar cells.^28^, ^29^ Currently, perovskite film format has been intensively researched and already produced high‐efficiency solar cells, while the study of perovskite single crystals is just in its infancy. Of great importance, single crystals are highly conducive to investigate fundamentally intrinsic properties of perovskites due to their absence of grain boundaries and existence of low trap density.^30^ Furthermore, it is well‐known that crystal shape and size of perovskites have profound impacts on their electrical and optical properties.

In this review article, we begin with discussing the crystallization and morphology control process of perovskite film formation, then turn to growth of large‐sized single crystals as well as free‐standing perovskite nanoparticles. Next, we highlight the dimensional evolution of perovskites from dimensions of three, two and one to zero dimension. Importantly, the characterization of optoelectronic properties of single crystals and their applications other than solar cells are summarized. Then, the growth mechanisms of these perovskite crystals are revealed. Perspectives for a fine control of perovskite structure and growth towards high‐performance electronic devices are finally outlined.

## 2 Film Formation of Perovskites

### 2.1 Chemical and Physical Depositions

**Figure 2:**
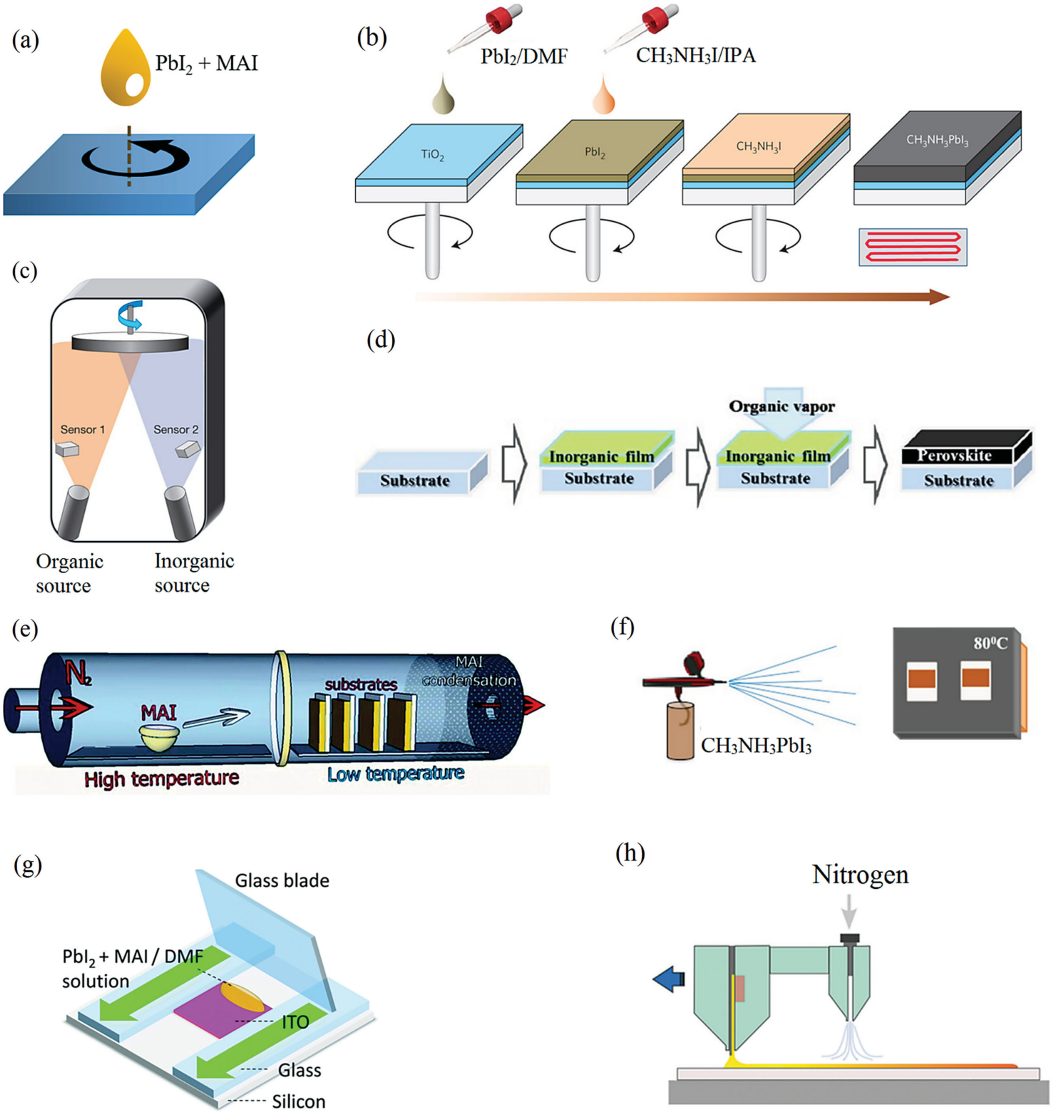
Schematic of perovskite film deposition methods. a) One‐step solution processing method. b) Two‐step spin‐coating procedure. Reproduced with permission.^34^ Copyright 2014, Nature Publishing Group. c) Dual‐source thermal evaporation system. Reproduced with permission.^8^ Copyright 2013, Nature Publishing Group. d) Vapor‐assisted solution process. Reproduced with permission.^35^ Copyright 2013, American Chemical Society. e) Hybrid chemical vapor deposition based perovskite synthesis. Reprinted with permission.^36^ Copyright 2014, Royal Society of Chemistry. f) Spray deposition technique. Reproduced with permission.^37^ Copyright 2015, American Chemical Society. g) Doctor‐blading. Reprinted with permission.^38^ Copyright 2015, Royal Society of Chemistry. h) Slot‐die coating with a gas‐quenching process. Reproduced with permission.^39^ Copyright 2015, Wiley‐VCH.

One advantage of organometal halide perovskites is their versatile processibility. As shown in **Figure** **2**, perovskite films can be prepared by chemical and physical deposition techniques, mainly including one‐step solution processing, two‐step sequential deposition, vapor deposition and vapor assisted solution processing.^31^, ^32^, ^33^ It is worth noting that perovskites film quality is susceptible to deposition conditions. Thus, it is of paramount importance to gain in‐depth knowledge of processing approaches, which enables further improvements. In the following discussion, we choose some representative examples to outline the above deposition methods.

One‐step solution processing is the simplest one among all the deposition methods. As shown in Figure 2a, this method is implemented by spin‐coating from a precursor solution of a mixture of Pb*X*_2_ and CH_3_NH_3_ *X* (*X* = Cl, Br, I) in a polar solvent such as γ‐butyrolactone (GBL), *N*,*N*‐dimethylformamide (DMF) or dimethylsulfoxide (DMSO).^6^, ^7^, ^40^, ^41^, ^42^, ^43^ Moreover, this method is one of the most possible ways for realizing the large‐area full‐printing manufacturing. For instance, flexible solar cells and photodetectors have been fabricated via this method.^44^, ^45^, ^46^

A two‐step deposition method was further developed to gain a better control of perovskite morphology. In this method, Pb*X*_2_ is firstly spin‐coated onto the substrate, followed by dipping or spin‐coating of CH_3_NH_3_ *X* perovskite film (Figure 2b).^34^, ^47^, ^48^, ^49^ This method was initiated by Burschka et al.^47^, in which PbI_2_ was first spin‐coated on a mesoporous TiO_2_ film and subsequently transformed into CH_3_NH_3_PbI_3_ by dipping it into a solution of CH_3_NH_3_I in 2‐propanol (IPA). The Park group later used a two‐step spin‐coating procedure to produce high‐quality perovskite films. It was found that the crystal size of as‐made CH_3_NH_3_PbI_3_ strongly depended on the concentration of CH_3_NH_3_PbI_3_.^34^ Generally, a lower concentration led to a bigger cuboid size.

Such a two‐step sequential deposition approach can be modified to obtain mixed halide perovskites. For example, morphology‐controllable CH_3_NH_3_PbI_3‐*x*_Cl*_x_* perovskites can be prepared by spin‐coating a mixed solution of CH_3_NH_3_Cl and CH_3_NH_3_I or a mixture of PbCl_2_ and PbI_2_.^50^, ^51^, ^52^ In another work, CH_3_NH_3_PbI_3‐*x*_Br*_x_* was synthesized through spin‐coating CH_3_NH_3_Br : CH_3_NH_3_I mixed precursor solution onto the PbI_2_ layers.^53^ Interestingly, pseudohalogen thiocyanate (SCN) was introduced into perovskite by spin‐coating a mixture of PbI_2_ and Pb(SCN)_2_ source in the first step, followed by coating a CH_3_NH_3_I layer.^54^ The final CH_3_NH_3_PbI_3‐*x*_(SCN)*_x_* perovskite films presented larger‐sized crystals and fewer traps than CH_3_NH_3_PbI_3_.

Vapor deposition method has also demonstrated as an effective way to deposit uniform and dense perovskite films. The Snaith group employed dual source evaporation system (i.e., separate CH_3_NH_3_I and PbCl_2_ sources) to deposit CH_3_NH_3_PbI_3‐*x*_Cl*_x_* films (Figure 2c).^8^ These films were extremely dense with crystal size of hundreds of nanometers, yielding a PCE of over 15%.

By combining current solution process and vacuum deposition, a novel low‐temperature vapor‐assisted solution process (VASP) was applied to fabricate perovskite films.^35^ The key step was in‐situ reaction of PbI_2_ film with CH_3_NH_3_I vapor, resulting in CH_3_NH_3_PbI_3_ films with full surface coverage and grain size up to microscale (Figure 2d). These VASP films based devices exhibited an impressive PCE of 12.1% within a planar architecture.

Chemical vapor deposition (CVD), as a cost‐effective way to scale up to industrial levels, was successfully applied to deposit perovskite films.^55^ For instance, the Qi group used hybrid CVD to synthesize perovskite films, where thermal‐evaporation of PbCl_2_ followed by vapor phase deposition of MAI (Figure 2e).^36^ A PCE as high as 11.8% was thus achieved. Noticeably, these cells exhibited decent stability and high reproducibility. The same group later demonstrated the CVD of FAPbI_3_.^56^ Later, aerosol‐assisted CVD, an ambient‐pressure CVD technique, was used to deposit perovskite films.^57^, ^58^ This method utilized the nebulization of precursor molecules, followed by transport of the aerosol in an inert carrier gas such as argon to a substrate surface where thermal decomposition of the precursors occurred. In another study, Lu and co‐workers fabricated uniform CH_3_NH_3_PbI_3_ films by a low‐pressure CVD technique, which effectively slowed down the over‐rapid intercalating reaction rate.^59^ The same team later demonstrated large‐area deposition of CH_3_NH_3_PbI_3_ films by in‐situ tubular CVD method.^60^

Besides CVD, spray‐coating is a viable processing protocol towards large‐area, low‐cost manufacture of perovskites. Figure 2f shows the schematic illustration of spray process. One‐step perovskite precursors are sprayed from a nozzle onto a hot substrate, followed by swift evaporation of solvent, thereby creating the perovskite crystals. For instance, Gamliel et al. fabricated thickness‐controllable CH_3_NH_3_PbI_3_ films by varying the number of spray passes in the spray process.^37^ In parallel, Barrows et al. spray‐coated CH_3_NH_3_PbI_3‐*x*_Cl*_x_* films with high surface coverage under ambient conditions after optimization of spraying parameters such as substrate temperature, casting solvent and post‐annealing temperature. As a result, a PCE of up to 11% was obtained.^61^ As another example, Das et al. applied ultrasonic spray‐coating of CH_3_NH_3_PbI_3‐*x*_Cl*_x_* on polyethylene terephthalate (PET) substrate to realize flexible perovskite solar cells with a PCE of 8.1%.^62^

Furthermore, other techniques such as blade‐coating^38^ and slot‐die^39^ were successfully applied to fabricate perovskite films, paving the way for low‐cost and large‐scale deployment of solar energy. For the blade‐coating process, the precursor solution was first dropped onto substrate, followed by a linear swipe using a glass blade with the relatively high speed (Figure 2g). By this means, the domain size of the bladed films can even reach 80–250 mm. For the slot‐die coating, PbI_2_ solution was first coated on substrate from one slot‐die head. Meanwhile, high‐pressure nitrogen from another head quickly dried the PbI_2_ film (Figure 2h). Perovskite film was therefore made by sequentially slot‐die coating of MAI solution. A PCE of 11.96% was achieved for slot‐die devices under ambient conditions.

### 2.2 Morphology Control

#### 2.2.1 Additives

Chloride was reported to have striking influences on the morphological evolution of perovskite thin films. In one‐step solution approach, standard CH_3_NH_3_PbI_3_ precursors—an equimolar mixture of CH_3_NH_3_I and PbI_2_—generally gives rise to poor surface coverage due to the fast crystallization of CH_3_NH_3_PbI_3_, which is thus unable to generate high‐efficiency solar cell devices.

In an initial attempt, Zhao et al. added CH_3_NH_3_Cl (MACl) to the standard CH_3_NH_3_PbI_3_ precursor solution and adjust the crystallization process for CH_3_NH_3_PbI_3_. The use of MACl led to the formation of pure CH_3_NH_3_PbI_3_ with enhanced absorption and improved coverage of CH_3_NH_3_PbI_3_ on planar substrate.^63^ MACl additive was also applicable to the reaction between PbBr_2_ and CH_3_NH_3_I for the fabrication of CH_3_NH_3_PbI_2_Br based PHJ solar cells.^64^

**Figure 3:**
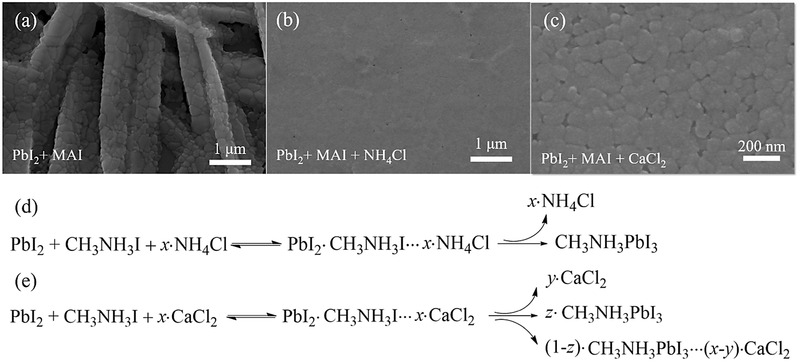
Top‐view SEM images of CH_3_NH_3_PbI_3_ thin films prepared from a) standard binary solution of CH_3_NH_3_I and PbI_2_, b) ternary mixture solution of PbI_2_, CH_3_NH_3_I, and NH_4_Cl without thermal annealing, and c) ternary mixture solution of PbI_2_, CH_3_NH_3_I, and CaCl_2_ without thermal annealing. Possible mechanisms in the formation of CH_3_NH_3_PbI_3_ film that is spin‐coated from d) ternary mixture solution of PbI_2_, CH_3_NH_3_I, and NH_4_Cl without thermal annealing and e) ternary mixture solution of PbI_2_, CH_3_NH_3_I, and CaCl_2_. a,b,d) adapted with permission.^65^ Copyright 2015, American Chemical Society. c,e) adapted with permission.^66^ Copyright 2015, Royal Society of Chemistry.

Inspired by that, Chen et al. further demonstrated a novel one‐step solution method of preparing perovskite films at room temperature. The non‐thermal fabrication of CH_3_NH_3_PbI_3_ films was realized by adding NH_4_Cl, a common cheap fertilizer, to the standard precursor solution of CH_3_NH_3_I and PbI_2_.^65^] **Figure** **3**a,b shows scanning electron microscopy (SEM) images to interpret the positive effects of NH_4_Cl on film morphological envolution. CH_3_NH_3_PbI_3_ film prepared from standard binary solution of CH_3_NH_3_I and PbI_2_ was discontinuous and consisted of elongated large crystal plates (Figure 3a). The NH_4_Cl strongly affected the crystallization process and optimized the film morphology of CH_3_NH_3_PbI_3_, yielding a flat and uniform perovskite film (Figure 3b). As displayed in Figure 3d, it seemed reasonable that adding NH_4_Cl to the standard precursor solution could lead to the rapid formation of an intermediate crystal structure like PbI_2_·CH_3_NH_3_I···*x*·NH_4_Cl. Excess NH_4_Cl could escape from intermediate due to the low vapor point of NH_4_Cl and spin‐induced low pressure in the film‐forming step.

In a subsequent report, NH_4_Cl was introduced to regulate crystallization of CH_3_NH_3_PbI_3‐*x*_Br*_x_*.^67^ In analogy to that of CH_3_NH_3_PbI_3_,^65^ the direct reaction between lead bromide (iodide) and methylamine iodide (bromide) usually led to a CH_3_NH_3_PbI_3‐*x*_Br*_x_* film with rough surface and low coverage. The incorporation of NH_4_Cl in the reaction system was found to effectively slow down the crystallization and provide more relaxation time, resulting in uniform and compact CH_3_NH_3_PbI_3‐*x*_Br*_x_* thin film regardless of the molar ratio between I and Br in the final compound. The optimal device based on CH_3_NH_3_PbI_2.4_Br_0.6_ led to a PCE of 12.1%. In another report, Wang et al. systematically studied the effect of chloride additives including NH_4_Cl, MACl and FACl on the film formation of HC(NH)_2_PbI_3_ (FAPbI_3_).^68^ The use of low‐volatility FACl and MACl assisted in the crystallization of black α‐FAPbI_3_ phase through the formation of non‐*δ*‐FAPbI_3_ intermediate phase, whereas NH_4_Cl led to the undesirable yellow *δ*‐FAPbI_3_ phase due to its highly volatile nature.

Despite these favorable influences upon addition of the above volatile chloride additives, the oppositely negative effects of nonvolatile chlorinated additives were revealed by Chen et al.^66^ As shown in Figure 3c, nonvolatile compound CaCl_2_ can significantly improve the film morphology of CH_3_NH_3_PbI_3_, which exhibited closely packed crystals and increased surface coverage. This regulation process was realized by an intermediate phase induced by the Cl^−^ inclusion. However, different than those volatile chlorinated additives, most Cl^−^ ions precipitated out, despite the partial inclusion into the CH_3_NH_3_PbI_3_ crystal, because insulating CaCl_2_ upon heat treatment remained in the final perovskite film (Figure 3e). As a result, CaCl_2_ treated CH_3_NH_3_PbI_3_ based planar solar cells were shown to yield poor device performance.

Inspired by the optimization of polymer film morphorlogy in OPVs, the Jen group chose an organic additive, 1,8‐diiodooctane (DIO), to modulate the crystallization rate and interfacial energy of CH_3_NH_3_PbI_3_, which yielded a best PCE of ≈12%.^69^ Incorporated DIO additive can chelate with Pb^2+^ in phase transformation, leading to distorted crystal lattice, increased internal energy and configurational entropy of growing crystals. As a result, crystals in DIO‐treated film displayed more regular faceting and improved interconnectivity than those of the pristine one. More recently, the same group further explored the feasibility of optimizing the molecular structures of alkyl halides additives to modulate crystallization of perovskite, since chain length and end‐group of these additives may affect intermolecular interactions between solvent and solute.^70^ Alkyl halides including 1,4‐diiodobutane (1,4‐DIB), 1,10‐diiododecane (1,10‐DID), 1,4‐dibromobutane (1,4‐DBrB) and 1,4‐dichlorobutane (1,4‐DClB) bearing different alkyl chain lengths and end‐groups have been systematically investigated to elucidate their influences on perovskite thin film evolution. It was found that C−*X* bond of alkyl iodides easily dissociated during the thermal annealing, which generated free *X* ^−^ ions to affect the coordination with Pb^2+^ in the perovskite formation. Consequently, these additives led to the formation of perovskite films with improved crystallization and better surface coverage, thereby giving a significant increase of PCE up to 13.1%. Another frequently used additive of OPV cells—1‐chloronaphthalene (CN)—was also applied to regulate the crystallization kinetics of perovskite, which improved both optical absorption and surface coverage than pristine film.^71^

In addition to alkyl halides, organic halide salts were found to be effective in improving the crystallinity and coverage of perovskite films. For instance, phosphonium halides with bulky aromatic cations, tetraphenylphosphonium iodide (TPPI) and chloride (TPPCl) successfully improved film morphology of perovskites and their device performance.^72^ The free halide ions from the salts may chelate to the Pb^2+^ cations and participate in the perovskite crystal formation, similar to the role of above‐mentioned alkyl halides, while the bulky and more rigid aromatic groups may alter the growth kinetic of the perovskite films. Moreover, TPPI was also an efficient interfacial n‐type dopant to reduce the contact resistance between PCBM and metal cathode. Consequently, PHJ solar cells of perovskites based on these phosphonium halides displayed an improved PCE up to 13%.

**Figure 4:**
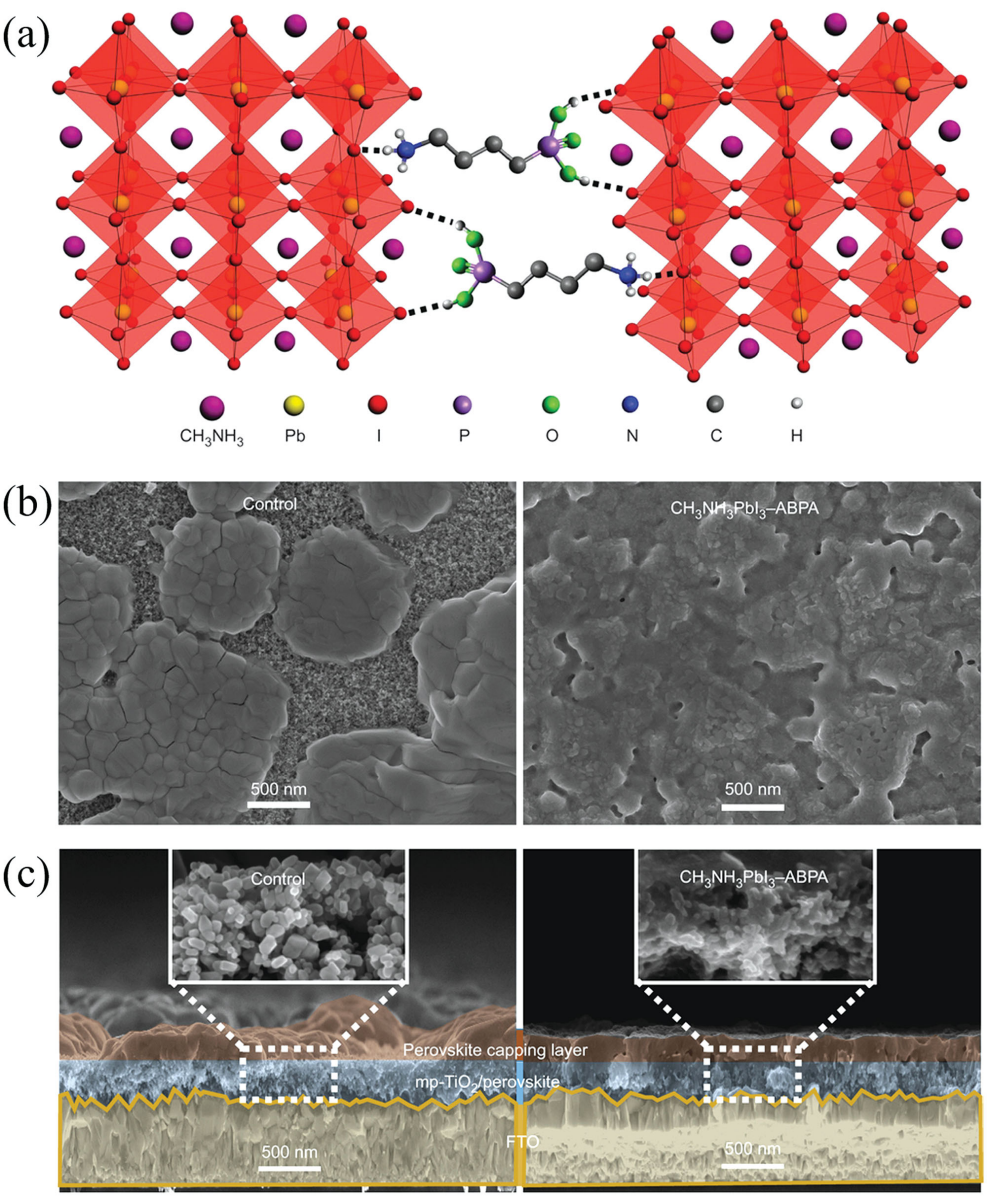
a) Schematic illustration of two neighboring grain structures in which the methyl ammonium groups are shown as one sphere for clarity, and the PbI_6_^4−^ octahedra are shown in red, crosslinked by butylphosphonic acid 4‐ABPACl hydrogen‐bonding interactions (O–H···I and N–H···I) of the iodide from the iodoplumbate complex with–PO(OH)_2_ and –NH^3+^ end groups of the 4‐ABPACl species. b) Surface and c) cross‐sectional SEM images of pristine (control) and 4‐ABPA‐anchored (CH_3_NH_3_PbI_3_–ABPA) perovskite films deposited on mesoscopic TiO_2_/FTO substrates by one‐step spin‐coating of the corresponding perovskite precursor solutions without or with 4‐ABPACl additive. a–c) Adapted with permission.^73^ Copyright 2015, Nature Publishing Group.

Very recently, the Grätzel group reported the use of butylphosphonic acid 4‐ammonium chloride (4‐ABPACl) as additive in a one‐step solution‐processing strategy, which resulted in stable and high‐performance perovskite solar cells.^73^ As shown in **Figure** **4**a, the terminal −NH^3+^ of 4‐ABPACl was inserted into empty A sites of perovskite through a supramolecular interaction, such as N–H···I^−^ hydrogen bonding. In the meantime, the other end group of −PO(OH)_2_ was strongly anchored to the perovskite surface via P–OH···I^−^ hydrogen bonding. As a consequence, the 4‐ABPACl molecules acted as crosslinking agents between neighbouring perovskite grains. This led to the formation of a smooth and uniform perovskite capping layer ranther than the rugged and discontinuous one without 4‐ABPACl additive (Figure 4b). Cross‐sectional SEM images in Figure 4c also indicated that the loading content of perovskite within mesoscopic TiO_2_ scaffold was remarkably increased with the additon of 4‐ABPACl. These two factors are combined to result in significant improvements of PV performance and moisture stability as opposed to pristine perovskites.

Long‐term stability of perovskite cells remains as the biggest hurdle prior to the commercialization. The use of ethylammonium iodide (EAI) additive can significantly improve the film quality and more importantly device stability.^74^ Devices based on EAI‐containing CH_3_NH_3_PbI_3‐*x*_Cl*_x_* perovskites retained approximately 80% of their pristine PCEs under accelerated heating (e.g., 65 °C) in a dark N_2_‐filled glove box for over 360 h. The enhanced stability largely arised from EAI suppressed crystallization of CH_3_NH_3_PbI_3‐*x*_Cl*_x_*. In a recent work, H_2_O additive was shown to be benificial for stability of CH_3_NH_3_PbI_3‐*x*_Cl*_x_*.^75^ The formation of CH_3_NH_3_PbI_3‐*x*_Cl*_x_*·nH_2_O hydrated perovskite during annealing process largely improved moisture resistance and thus device stability. In another report, the water content (≤10 vol%) in perovskite precursors was found to have insignificant influence on the photovoltaic performance of devices.^76^

More recently, polymer additives have demonstrated as a simple yet novel strategy to optimize the film morphology of perovskites.^77^ For instance, Su et al. reported that poly(ethylene glycol) (PEG) additive can not only facilitated CH_3_NH_3_PbI_3‐*x*_Cl*_x_* precursor solution to spread out smoothly, but also retarded the growth and aggregation of perovskite crystals, both of which resulted in dense and smooth films. However, the main drawback for such polymer additive was its insulating characteristics, which caused unfavorably an increase of series resistance. Therefore, the amount of addition must be carefully controlled; in the above report, a 25% increase in PCE was achieved by adding 1 wt% of PEG additive.

Spin‐coating of a mixed solution of formamidinium iodide (FAI) and PbI_2_ precursors in DMF initially yielded discontinuous perovskite films. To resolve this issue, the Snaith group added a small amount of hydroiodic acid (HI) to the stoichiometric FAI : PbI_2_ (1 : 1 mol%) precursor solution.^78^ Such added acid promoted the solubilization of the inorganic component and hence hindered the FAPbI_3_ crystallization, generating extremely uniform and continuous film with high phase purity. The same group later employed HI additive to successfully stabilize inorganic Caesium lead iodide (CsPbI_3_) perovskites over 300 °C.^79^ In a following report, HI additive was added to produce pinhole‐free MAPbI_3_ perovskite thin film with high phase purity.^80^ The added HI recovered the methylamine, a decomposed product of MAI, into MAI, thereby significantly suppressing the decomposition reaction of MAPbI_3_ perovskite. Consequently, the formation of PbI_2_ impurities was notably inhibited whereas conventional MAPbI_3_ in DMF or DMSO solution made some PbI_2_ impurity inevitably. Finally, the HI‐treated device exhibited a high PCE of 17.2% irrespective of the scan direction and rate.

In two‐step deposition method, additives have also demonstrated to play a crucial role. As is well‐known, the PbI_2_ morphology is a prerequisite for high‐quality perovskite thin film in two‐step sequential deposition. Therefore, additives such as acid, water and DMF were generally utilized in the first‐step PbI_2_ precursor solution. For instance, Leung et al. successfully prepared uniform and continuous MAPbI_3_ thin film by adding HCl into PbI_2_ precursor solution.^81^ The addition of HCl inhibited the formation of rod‐shape PbI_2_ and promoted homogeneous nucleation and crystal growth. The HCl‐treated device displayed a respectable PCE of 15.2% with remarkably improved environmental stability. Zhao et al. also fabricated pinhole‐free planar CH_3_NH_3_PbI_3_ perovskite film with the aid of HCl in both one‐step and two‐step deposition methods.^82^ Importantly, these films exhibited high humidity tolerance, which were stable even under relative humidity (RH) of 60%. Likewise, a small amount of H_2_O was added into PbI_2_ in DMF solution to make a homogenous precursor solution, giving rise to high‐quality PbI_2_ film with full surface coverage.^83^ Based on such PbI_2_ film, the resulting MAPbI_3_ perovskite film was highly pure, smooth and very dense even without any pinhole.

Moreover, the use of MAI additive in the first step of sequential deposition can affect the morphology of PbI_2_ raw film and hence final MAPbI_3_ film.^84^, ^85^ Upon MAI addition, the volume of PbI_2_ pre‐expanded and crystallinity reduced, which favored the complete conversion to MAPbI_3_ in the second step.^85^ Later, Zhao et al. further developed a three‐step sequential solution process to prepare PbI_2_‐free CH_3_NH_3_PbI_3_ perovskite films by adding MACl additive in PbI_2_ precursor solution.^86^ A thermally unstable film made of stoichiometric PbI_2_ + CH_3_NH_3_Cl precursors was first deposited, followed by thermal decomposition, giving rise to PbI_2_. This way accelerated the conversion of PbI_2_ to CH_3_NH_3_PbI_3_ without any traceable PbI_2_ residues when dipping into CH_3_NH_3_I solution, thus improving the device performance.

**Table** **1** summarizes all above‐mentioned additives and their corresponding perovskite systems. It has been manifested that chlorides including HCl, NH_4_Cl, MACl, FACl, TPPCl and 4‐ABPACl play an imaportant role in the formation of highly uniform film, although the question whether the chloride ions enter into perovskite crystal lattices is still under hot debate. Moreover, given the use of HI, MAI, EAI, TPPI, 1,4‐DIB, 1,10‐DID and DIO, it seems reasonable that iodide ion, to some extent, aids to form high‐quality perovskite film. All these results indicate that I^−^ and Cl^−^ anions may chelate to Pb^2+^ cations and alter the kinetic of perovskite crystallization. Moreover, the volatility of additives should been taken into account for their selection, as seen from the examples of NH_4_Cl, CaCl_2_, MACl, and FACl. Lastly, in addition to DIO and CN, other additives that are used in OPVs can be employed to regulate the crystallization of perovskites.

**Table 1:** Representative additives and their corresponding perovskite systems

Additives	Systems	Additives	Systems
H_2_O	One‐step, MAPbI_3‐*x*_Cl*_x_* 75	HI	One‐step, FAPbI_3_ 78
	two‐step, MAPbI_3_ 83		One‐step, CsPbI_3_ 79
HCl	one‐step and two‐step MAPbI_3_ 81, 82		One‐step, MAPbI_3_ 80
	One‐step, MAPbI_3_ 65		
NH_4_Cl	One‐step, MAPbI_3‐*x*_Br*_x_* 67	MAI	Two‐step, MAPbI_3_ 84, 85
	One‐step, MAPbI_3_ 63		
MACl	One‐step, MAPbI_2_Br^64^	EAI	One‐step, MAPbI_3‐*x*_Cl*_x_* 74
	One‐step, FAPbI_3_ 68	1,4‐DIB, 1,10‐DID, 11,4‐DBrB and 1,4‐DClB	One‐step, MAPbI_3‐*x*_Cl*_x_* 70
	Two‐step, MAPbI_3_ 86		
FACl	One‐step, FAPbI_3_ 68	DIO	One‐step, MAPbI_3_ 69
TPPI and TPPCl	One‐step, MAPbI_3‐*x*_Cl*_x_* 72	CN	One‐step, MAPbI_3‐*x*_Cl*_x_* 71
4‐ABPACl	One‐step, MAPbI_3_ 73	PEG	One‐step, MAPbI_3‐*x*_Cl*_x_* 77

#### 2.2.2 Thermal Annealing

Thermal annealing is required for most of the above‐mentioned deposition techniques to remove residual solvents or additives and hence crystallize perovskites in thin film. Arguably, thermal annealing process is found to critically impact film formation of perovskites and thus their photovoltaic performance.

Thermal annealing can induce chemical and structural changes in the perovskite layer. An interesting work was done by Wiesner et al. showing that solution‐processed perovskite films underwent the evolutions between three distinct crystalline structures during thermal annealing—a crystalline precursor structure not described previously, a 3D perovskite structure, and a mixture of compounds resulting from degradation.^87^ Crystalline precursors went through a solid‐solid phase transformation to the 3D perovskite structure at 80 °C. This transition was consistent with Ostwald′s ‘Rule of Stages' in which a metastable precursor is first formed and then transformed into the more stable product. In another study, it was shown that the ratio between the MA and FA cations might change in a mixed film of MAPbI_3_ and FAPbI_3_ during thermal annealing process, which affected both band gap and stability of the layers.^88^ Niwano and colleagues later found that upon thermal annealing, both CH_3_NH_3_PbCl_3_ and CH_3_NH_3_PbI_3_ crystals in mixed‐halide perovskite layer were initially formed from an amorphous phase.^89^ Such amorphous phase and MAPbCl_3_ crystals served as ions supply for the growth of MAPbI_3_ crystals when the MA and chloride ions were evaporated from the mixed‐halide perovskite layer, resulting in a thin layer composed of large MAPbI_3_ grains.

Annealing temperature is of prime importance in the perovskite film formation. In an early attempt, the Grätzel group investigated its effect on film morphology and composition, and then correlated it with photovoltaic performance and working mechanisms of device.^90^ It was found that annealing temperature of 80 °C was required to fully form CH_3_NH_3_PbI_3_ perovskites. On the one hand, lower temperature led to the removal of solvent yet incomplete conversion of CH_3_NH_3_PbI_3_ crystals, suggesting that the reaction between PbCl_2_ to and CH_3_NH_3_I was endothermic. On the other hand, too high temperature led to the formation of PbI_2_, which was detrimental to device performance. The optimal annealing temperature was thus determined in the range of 80−100 °C, giving rise to the film morphology consisting of interconnected network of perovskite crystallites without additional PbI_2_, which showed the best device performance.

Later, the Snaith group found that a rapid annealing at 130 °C induced the growth of micron‐sized perovskite crystal domains while a long yet moderate annealing at 100 °C resulted in polycrystalline domains with size of 100−1000 nm.^91^ However, these two thermal processing protocols had different influences on photovoltaic performances of MSSC and PHJ architectures. For the MSSC devices, the 100 °C annealed samples performed better than 130 °C annealed ones because of their higher perovskite surface coverage. Conversely, for PHJ cells, the 130 °C annealed samples outperformed the 100 °C treated counterparts due to their highly textured morphology. This work highlighted that simultaneous control of macroscopic morphology and crystalline domain size was of capital importance in thermal annealing.

In addition to annealing temperature, thermal annealing time was found to have great impacts on film morphology of perovskites.^92^ A thermal annealing at 105 °C for 15 min was efficient enough to drive the formation of phase‐pure perovskites. When thermal annealing time prolonged to 2 h, the crystallization and gain size of perovskites were increased without losing film continuity or coverage, thus leading to remarkably enhanced charge mobility and significantly increased fill factor (FF) and short‐circuit current density (*J*_sc_). However, a longer time annealing of 3 h led to perovskite decomposition to PbI_2_ phase.

In order to avoid poor film morphology caused by fast evaporation of solvent and decomposition of perovskite, a controllable scheme of gradual annealing at low temperature was utilized to replace traditional isothermal annealing method.^93^ In this scheme, annealing temperature was increased gradually from a low temperature value, which favored the formation of highly crystalline perovskite films with homogenous surface coverage and micrometer‐level diffusion lengths. A PCE of 15% was reached for a planar perovskite solar cell fabricated from the optimal gradual annealing process. Such a stepwise ramp annealing was also found to be correlated with solvent evaporation rate, which was particularly critical for the nucleation and growth in order to yield high surface coverage.^94^

Recently, a hot‐casting technique was developed as a useful means of thermal treatment to grow dense perovskite films with millimeter‐scale grains.^95^ In this method, a hot solution (≈70 °C) of perovskite precursor was spin‐casted onto substrate with a stable temperature of 180 °C. Such a high temperature provided enough thermal energy for the growth of perovskite crystals to follow a Volmer−Weber mode.^96^ The precursor solution was first solidified and then transformed into pure crystalline perovskite films with no intermediate phase in a few seconds, which circumvented additional thermal annealing. Planar solar cells based on such hot‐casted perovskite films showed a PCE of 18% with no hysteresis, attributable to reduced bulk defects and improved charge carrier mobility in large grains.

#### 2.2.3 Solvent Annealing

Complementary to thermal‐annealing, solvent annealing where solvent vapor is introduced during the perovskite crystallization was found to be an effective method of increasing grain size and crystallinity of perovskites. DMF (the most commonly used solvent for perovskite) vapor annealing enables to solubilize the solid film, which allows for the precursor ions or molecules to diffuse a longer distance than thermal annealing. For instance, the Huang group fabricated solvent‐annealed MAPbI_3_ film with an average grain size of increased up to 1 μm, which is comparable to the film thickness, largely exceeding the maximum grain size (≈260 nm) in thermally annealed films.^97^ Moreover, the solvent annealed film exhibited dramatically reduced trap density, increased charge recombination lifetime, decreased charge extraction time, and increased carrier diffusion length, all of which significantly enhanced the device performance. Besides DMF, vapor annealing under DMSO solvent or DMF/chlorobenzene mixed solvents has proved effective in improving perovskite crystallization for high performance solar cells.^98^, ^99^

By combining solvent annealing and thermal annealing, high‐quality perovskite film was controllably obtained by Feng et al.^100^ Solvent annealing in DMF vapor was first applied to promote migration and interdiffusion of the solvent‐assisted precursor ions and molecules, which resulted in large‐sized grain growth. The subsequent thermal annealing further improved film crystallization and morphology. Perovskite films fabricated from such two‐step annealing process exhibited high uniformity, large grain size up to 1.1 μm, and highly preferred growth orientation along the (110) direction, collectively yielding an significantly enhanced PCE of 14%. Such two‐step solvent‐thermal annealing method offers a facile and effective strategy to obtain high‐quality perovskite film.

#### 2.2.4 Atmospheric Effects

Organometal halide perovskites are susceptible to the environment and therefore an appropriate control of atmosphere including oxygen and moisture can facilitate its crystallization. Thermal annealing in O_2_ was found to substantially increase PCE, which can be explained by the fact that O_2_ diffusion can help reduce defect density at the grain boundaries and within the bulk of perovskites.^101^, ^102^ In addition, if the hole transporting layer is spiro‐MeOTAD, O_2_ treatment would cause p‐type doping and thus increase its electrical conductivity.

Moisture‐assisted crystal growth has demonstrated as an effective way to improve the film quality, grain size, carrier mobility, and lifetime of perovskites.^103^ Owing to the high hydrophilicity of CH_3_NH_3_I, an exposure to moderate moisture of relative humidity (RH = 35 ± 5%) would cause the perovskite grain boundary to creep and subsequently merge adjacent grains, generating larger crystal grains. Meanwhile, perovskite underwent recrystallization, which enlarged the diffusion length and yielded better quality of perovskite film. More recently, Zou et al. reported that spin‐coating under low RH, accompanied by thermal annealing under high RH, were beneficial for increasing perovskite crystallinity and improving device performance.^104^ At the spin‐coating stage, low RH led to the formation of high nucleation density, leading to in‐plane layer growth and hence high coverage of perovskite films. Yet at the subsequent thermal annealing stage, the modest supersaturation induced by high RH benefited the formation of perovskite films with good crystallinity. Most recently, it was found that hydrated perovskite crystalline phases formed during its exposure to water vapor at room temperature.^105^ This hydration process was reversible in the absense of condensed water.

In another study, both oxygen and moisture were found to greatly affect the luminescent properties of perovskites.^106^ The influence of oxygen was predominant during the photo‐activation process while moisture played a leading role in the photo‐darkening process. Oxide and hydrate species which resulted from the reactions between photogenerated carriers and atmosphere were likely the main reason for the observed phenomena.

Very recently, it was demonstrated that perovskite films prepared by ambient annealing exhibited comparable solar cell performance to those annealed in dry N_2_.^107^, ^108^ The formation of MAPbCl_3_ phase was inhibited when annealing in air with RH of 50%, which favored MAPbI_3_ crystallization. As a result, thermal annealing in air led to a significant increase of both crystallinity and crystal size of CH_3_NH_3_PbI_3_ as compared to that in nitrogen.^109^

#### 2.2.5 Solvent Engineering

Solvent engineering technique has proved as a simple yet effective means for realizing high‐performance perovskite solar cells. Generally, DMF, DMSO, GBL, and *N*‐methyl‐2‐pyrrolidone (NMP) are good solvents for lead halides and MAI while chlorobenzene, benzene, xylene, toluene, 2‐propanol, and chloroform are poor solvents for perovskites. Thus, the most commonly used solvent‐engineering strategy is the utilization of either mixed solvents or/and anti‐solvents, which leads to favorable perovskite film morphology.^110^, ^111^

For instance, extremely uniform and dense CH_3_NH_3_Pb(I_1−*x*_Br*_x_*)_3_ perovskite layers with grain sizes in the range of 100−500 nm were obtained by using a mixed solvent of GBL and DMSO, followed by drop‐casting of toluene during spin‐coating process.^112^ It is thus plausible that the formation of a stable MAI(Br)‐PbI_2_‐DMSO phase via an intercalation process played a vital role in impeding the reaction between MAI(Br) and PbI(Br)_2_.

**Figure 5:**
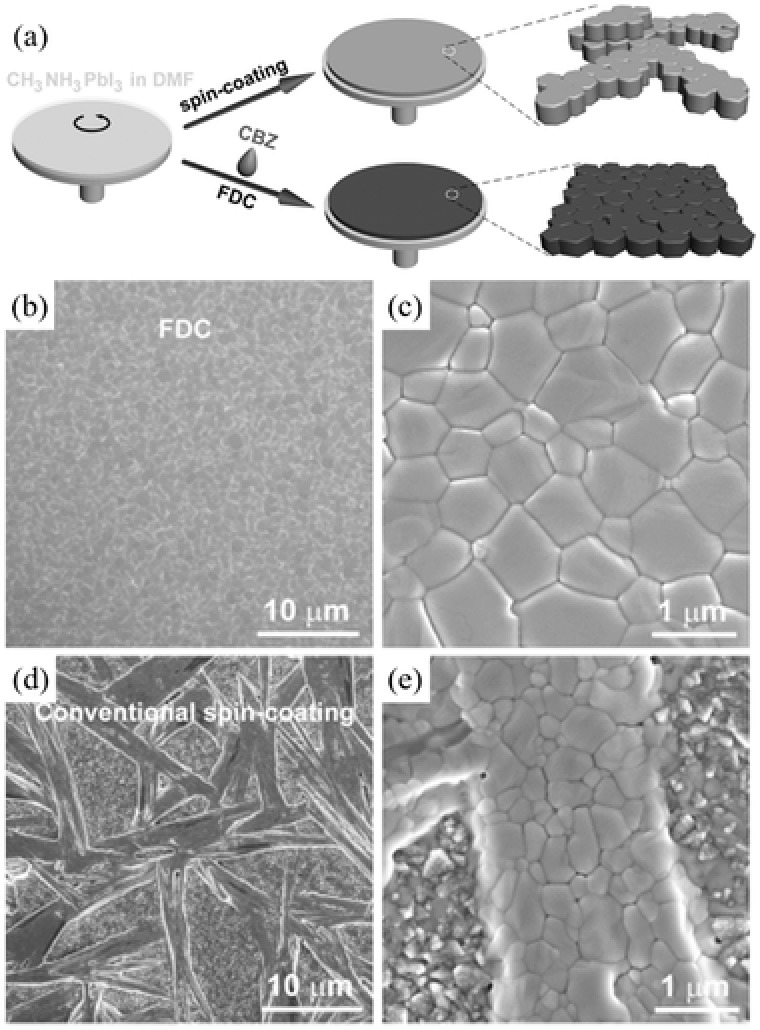
a) Schematic illustration of the FDC process and conventional spin‐coating process for fabricating perovskite films. Low‐ and high‐magnification SEM images of the surface of a CH_3_NH_3_PbI_3_ film prepared b,c) by FDC with the addition of chlorobenzene and d,e) by conventional spin‐coating. a–e) Adapted with permission.^113^ Copyright 2014, WILEY‐VCH.

As another example, a fast‐deposition crystallization (FDC) procedure was developed to yield highly uniform perovskite film consisting of micro‐sized crystals, as illustrated in **Figure** **5**a.^113^ In this process, a DMF solution of CH_3_NH_3_PbI_3_ was first spin‐coated on the substrate, immediately followed by exposure to a second solvent, which is usually poor solvent for perovskites, such as chlorobenzene. An instant darking of the film was observed, indicating the fast crystallization of perovskite in FDC. This phenonmenon can be attributed to rapidly reduced solubility of CH_3_NH_3_PbI_3_ and thus fast nucleation and growth of CH_3_NH_3_PbI_3_ upon the drop of second solvent. As displayed in Figure 5b,c, perovskite film fabricated by FDC yielded micron‐sized grains and full surface coverage. In contrast, conventional spin‐coating process leaves wet film to dry slowly and hence a shiny‐gray film of perovskite was obtained. Subsequent thermal annealing at 100 °C for 10 min is required to remove residual solvent and further promote crystallization. This film however exhibited large rod‐like grains with an incomplete coverage (Figure 5d,e).

Very recently, Zhou et al. proposed solvent–solvent extraction concept for room‐temperature processing of high‐quality perovskite thin films.^114^ After spin‐coating of perovskite precursor solution in a high‐boiling‐point solvent (e.g., NMP), the wet film was immediately immersed in a bath of diethylether, a low‐boiling‐point solvent. This way efficiently extracted NMP solvent and meanwhile induced a rapid crystallization of ultra‐smooth perovskite films.

## 3 Perovskite Single Crystals

### 3.1 Shape Evolution

Besides polycrystalline thin films, single crystals are another main form of perovskites. It is known that the crystal lattices of single crystals are continuous and unbroken. Importantly, there are no grain boundaries in perovskite single crystals, resulting in fewer defects than polycrystalline analogues. Moreover, this monocrystalline nature imparts them with unique optical and electrical properties, which depends on the size and shape of crystallites. Therefore, a good understanding and fine control of single crystal growth of perovskites could further boost their optoelectronic performance. In the following discussion, we will summarize shape evolution of perovskite single crystals ranging from 3D large sized single crystals, 2D nanoplates and 1D nanowires to zero‐dimensional (0D) quantum dots. In particular, their growth mechanisms are unraveled with a focus on the correlation between their crystallographic structures and optoelectronic characteristics.

#### 3.1.1 3D Large‐Sized Single Crystals

Recently, large‐size perovskite single crystals have drawn intensive interest owing to their remarkably lower trap densities, higher charge mobilities and longer carrier diffusion lengths than their polycrystalline thin film counterparts.^115^, ^116^ It was therefore projected that perovskite single crystals hold huge potential to further boost the PCE up to 25% in solar cells.^30^ In order to gain a deep insight into fundamental properties of perovskites, it is of prime importance to generate phase‐pure single crystals.

**Figure 6:**
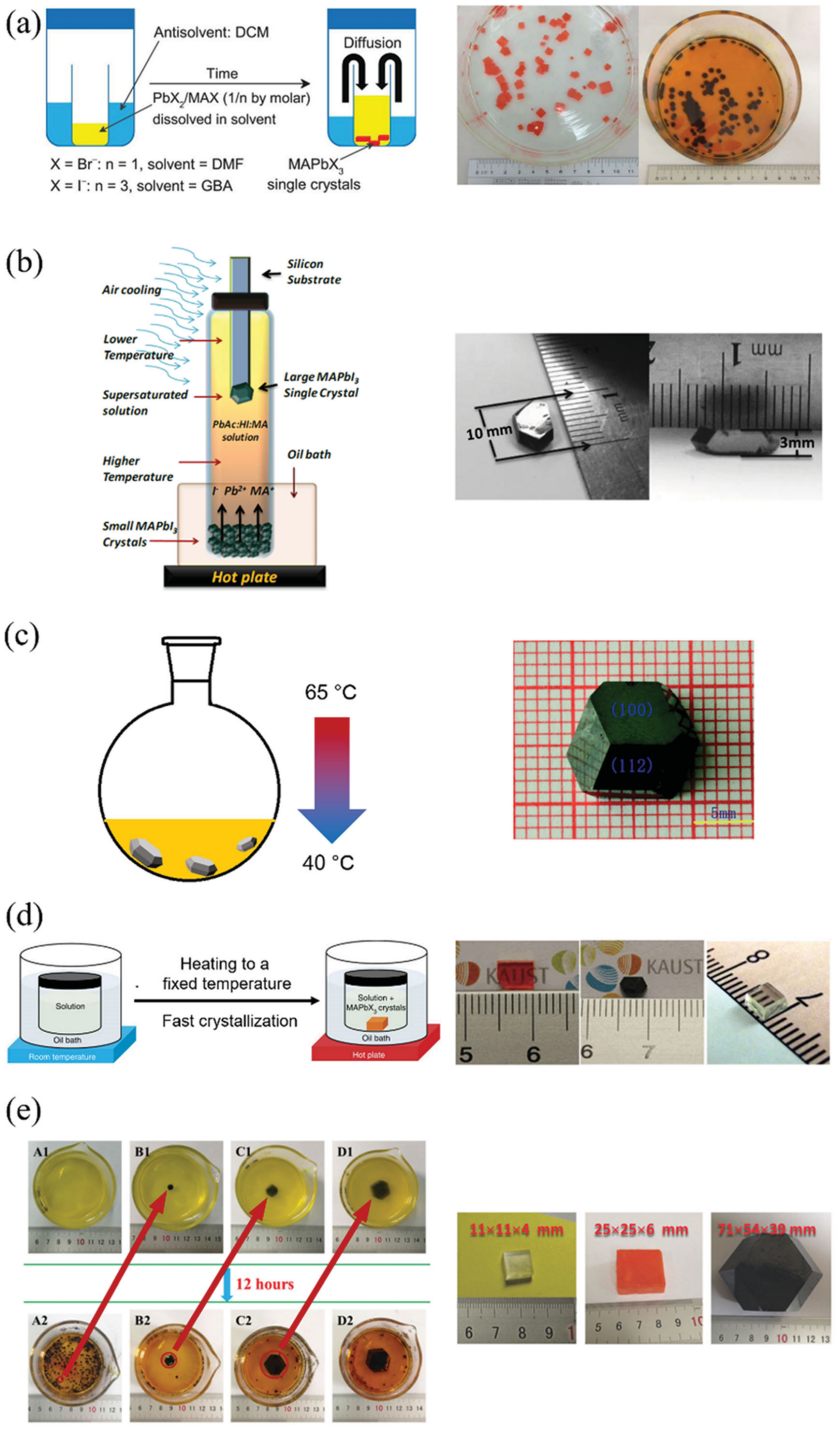
Schematic of growth methods of large‐sized single crystals and the corresponding photographs of as‐grown single crystals. The color of black refers to MAPbI_3_, red MAPbBr_3_ and white MAPbCl_3_. a) Antisolvent vapor‐assisted crystallization method. Adapted with permission.^115^ Copyright 2015, American Association for the Advancement of Science. b) Top‐seeded solution‐growth method. Adapted with permission.^116^ Copyright 2015, American Association for the Advancement of Science. c) Cooling‐induced crystallization method. Adapted with permission.^117^ Copyright 2015, Royal Society of Chemistry. d) Inverse temperature crystallization apparatus. Left part: Adapted with permission.^118^ Copyright 2015, Nature Publishing Group. Right part: Adapted with permission.^119^ Copyright 2015, American Chemical Society. e) Repeated seed‐solution growth method. Adapted with permission.^120^ Copyright 2015, Wiley‐VCH.

By far, the most common cultivation methods of producing 3D large‐sized perovskite single crystals include antisolvent vapor‐assisted crystallization, seed solution‐growth method, cooling‐induced crystallization and solvothermal growth. **Figure** **6** shows schematic diagrams of these typical growth methods and photographs of their corresponding as‐grown single crystals. For instance, growth of millimeter‐sized crack‐free CH_3_NH_3_PbX_3_ single crystals were achieved by using an antisolvent vapor‐assisted crystallization method, in which dichloromethane was slowly diffused into CH_3_NH_3_PbX_3_ precursor solution in DMF (Figure 6a).^115^ These high‐quality single crystals exhibited exceptionally low trap‐state densities of ≈10^9^−10^10^ cm^‐3^ and incredibly long charge carrier diffusion lengths exceeding 10 μm.

**Figure 7:**
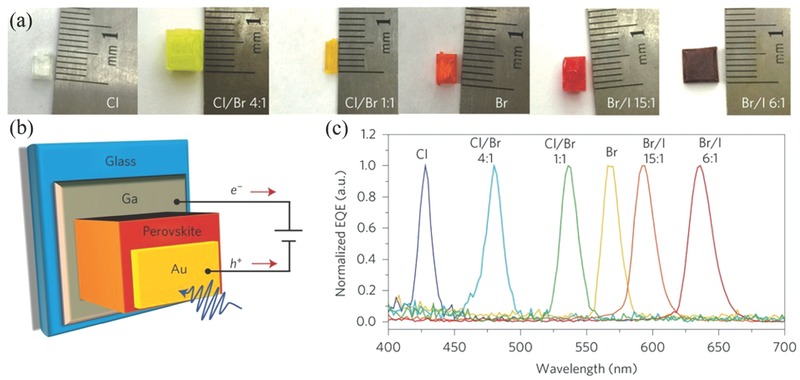
a) Photographs of single‐halide and mixed‐halide perovskite single crystals with different halide compositions. b) Schematic of photodetector structure. c) Normalized EQE spectra of the single‐halide and mixed‐halide perovskite single‐crystal photodetectors with different halide compositions, showing the ultranarrow EQE peak and tunable spectral response. The EQE spectra were measured under −1 V bias. a–c)Reproduced with permission.^121^ Copyright 2015, Nature Publishing Group.

In a recent report, the Huang group grew millimeter‐sized CH_3_NH_3_PbI_3_ single crystals from a supersaturated solution by using a top‐seeded solution‐growth method with a temperature gradient.^116^ As shown in Figure 6b, small‐sized single crystals served as ions supply for a saturated solution in the bottom of container. Meanwhile, the cooler top‐half solution was supersaturated to produce large‐sized single crystals. The small temperature difference between the bottom and top solutions provided sufficient driving force for the growth of large crystals. Interestingly, the diffusion length of CH_3_NH_3_PbI_3_ single crystals exceeded 175 μm under 1 sun light illumination and even surpassed 3 mm under 0.003% sun irradiation, which arised from higher carrier mobility, longer lifetime and largely suppressed trap densities in single crystals than those of polycrystalline thin films. More intriguingly, the internal quantum efficiencies (IQEs) of 3 mm‐thick single crystal approached 100% under weak light. The same group later successfully utilized single crystals as the photoactive materials in photodetectors, which possessed very narrow band photodetection.^121^ Single‐ and mixed‐halide perovskite single crystals with different Cl/Br and Br/I precursor ratios were thus implemented by cooling‐induced crystallization method. Photographs of these single crystals are presented in **Figure** **7**a. For MAPbBr_3−*x*_Cl*_x_* single crystals, the color gradually varied from transparent to yellow and finally to orange when molar ratio of Br/(Cl + Br) increased from 0 to 1 in the precursor solution. For MAPbI_3−*x*_Br*_x_* single crystals, on the other hand, the color progressively changed from orange to red with increasing molar ratio of I/(I + Br). Photodetectors based on these single crystals were constructed as shown in Figure 7b. The thickness of the single crystals in these devices was about 1 mm. Thin layers of Au and Ga were used as the semi‐transparent anode and cathode, respectively. Under light illumination, free electrons and holes were generated near the Au electrode, and then drifted across the single crystals towards the Ga and the Au, respectively, under the applied electric field. Output photocurrent was finally generated after the collection of these electrons and holes. Figure 7c shows the normalized external quantum efficiency (EQE) spectra of these perovskite single‐crystal photodetectors. The response spectrum can be continuously tuned from blue to red by varying the halide composition in single crystals. All the devices exhibited a single narrow peak, which was distinct from the wide spectra observed in the thin‐film perovskite photodetectors. Moreover, the full‐width at half‐maximums (FWHMs) of single‐crystal photodecttors were very narrow (less than 20 nm), holding good prospect for applications such as selected‐wavelength imaging, flame detection and so on.

Tetragonal CH_3_NH_3_PbI_3_ single crystals with dimensions of 10 mm × 10 mm × 8 mm were grown by a cooling‐induced crystallization method in HI solution.^117^ A decrease of temperature from 65 to 40 °C caused a saturation of the solute and hence the growth of CH_3_NH_3_PbI_3_ crystals (Figure 6c). The band gap of CH_3_NH_3_PbI_3_ single crystals was ca. 1.48 eV, which was smaller than those derived from polycrystalline thin films. Moreover, CH_3_NH_3_PbI_3_ single crystals exhibited a relatively good thermal stability, holding good promise for optical and electronic applications. In another work, Zhao et al. prepared mixed halide perovskite CH_3_NH_3_Pb(Br_1‐*x*_Cl*_x_*)_3_ single crystals from stoichiometric PbBr_2_ and [(1‐*y*) CH_3_NH_3_Br +*y* CH_3_NH_3_Cl] precursor solutions in DMF by a facile solvothermal growth method.^122^ The Cl/Br ratio in single crystals is larger than that of pristine precursor solution, unveiling an unusual mechanism of crystal growth. It was also suggested that Cl and Br exhibited different affinities of forming CH_3_NH_3_Pb(Br_1‐*x*_Cl*_x_*)_3_ single crystals. Moreover, with the increase of Cl content, the band gap of single crystals increased while the unit cell dimensions decreased. It was also found that the formation of CH_3_NH_3_PbBr_3_ single crystals did not result from the evaporation of DMF, which was different from that using other crystal growth methods. Thus, crystallization of CH_3_NH_3_PbBr_3_ in DMF could be an endothermic reaction. Later, Chang and co‐workers grew CH_3_NH_3_PbBr_3_ single crystals with a size of 14 × 14 mm by using a simple single‐solution fabrication method.^123^ It is interesting to note that a large change of surface potential by 200 mV was observed in CH_3_NH_3_PbBr_3_ single crystals, which was presumably caused by the light‐enhanced ‘polarization effect' in CH_3_NH_3_PbBr_3_ lattices.

However, the above solution crystallization processes for perovskite single crystals were subject to slow growth rate. Thus, the Bakr group developed a rapid crystal growth approach—inverse temperature crystallization (ITC)—to obtain CH_3_NH_3_PbX_3_ single crystals with size and shape controllability at a rate that was an order of magnitude faster than that using the previously reported growth methods.^118^ DMF and GBL were chosen for ITC growth of MAPbBr_3_ and MAPbI_3_ single crystals, respectively, due to their substantial drop of solubility in the corresponding solvents with increasing temperature. As shown in Figure 6d, by setting a high temperature of the heating bath, single crystals quickly participated out. Despite their fast growth rates, both MAPbBr_3_ and MAPbI_3_ single crystals exhibited charge transport characteristics comparable to those grown by conventional techniques of cooling or antisolvent vapor‐assisted crystallization. The same group later applied ITC method to grow sizable CH_3_NH_3_PbCl_3_ single crystals through judicious selection of DMSO−DMF co‐solution.^119^ The grown CH_3_NH_3_PbCl_3_ crystals displayed a sharp absorption edge at 435 nm and a strong photoluminescence (PL) peak at 440 nm, making them attractive candidates for visible‐blind UV photodetector applications. Such ITC method also led to the successful growth of FAPbX_3_ single crystals.^124^ By combining the above cooling solution and ICT methods, a large 5 mm‐sized FAPbI_3_ single crystal was obtained, which exhibted a long carrier lifetime of 484 ns, a high carrier mobility of 4.4 cm^2^ V^−1^s^−^^1^, and a conductivity of 1.1 × 10^−7^ (Ω cm)^−1^.^125^

Interestingly, two‐inch‐sized CH_3_NH_3_Pb*X*_3_ (*X* = Cl, Br, I) crystals were grown from a repeated seed‐solution growth method.^120^ As displayed in Figure 6e, small particulates in 2 mm‐diameter were first selected as seed crystals in percursor solution at 100 °C for the growth of larger crystals. The obtained large crystal was further used as the new seed. By repeating these steps, the largest crystal can even reach a size of 71 mm × 54 mm × 39 mm. CH_3_NH_3_PbBr_3_ and CH_3_NH_3_PbI_3_ single crystals possessed a low trap density of 10^9^−10^10^ cm^−3^, a high carrier mobility of 4.36−34 cm^2^ V^−1^ s^−1^ and good thermal stability, holding strong promise for high‐performance optoelectronic applications. In addition, it was found that CH_3_NH_3_PbCl_3_ single crystals displayed a PL emission at 402 nm.

**Figure 8:**
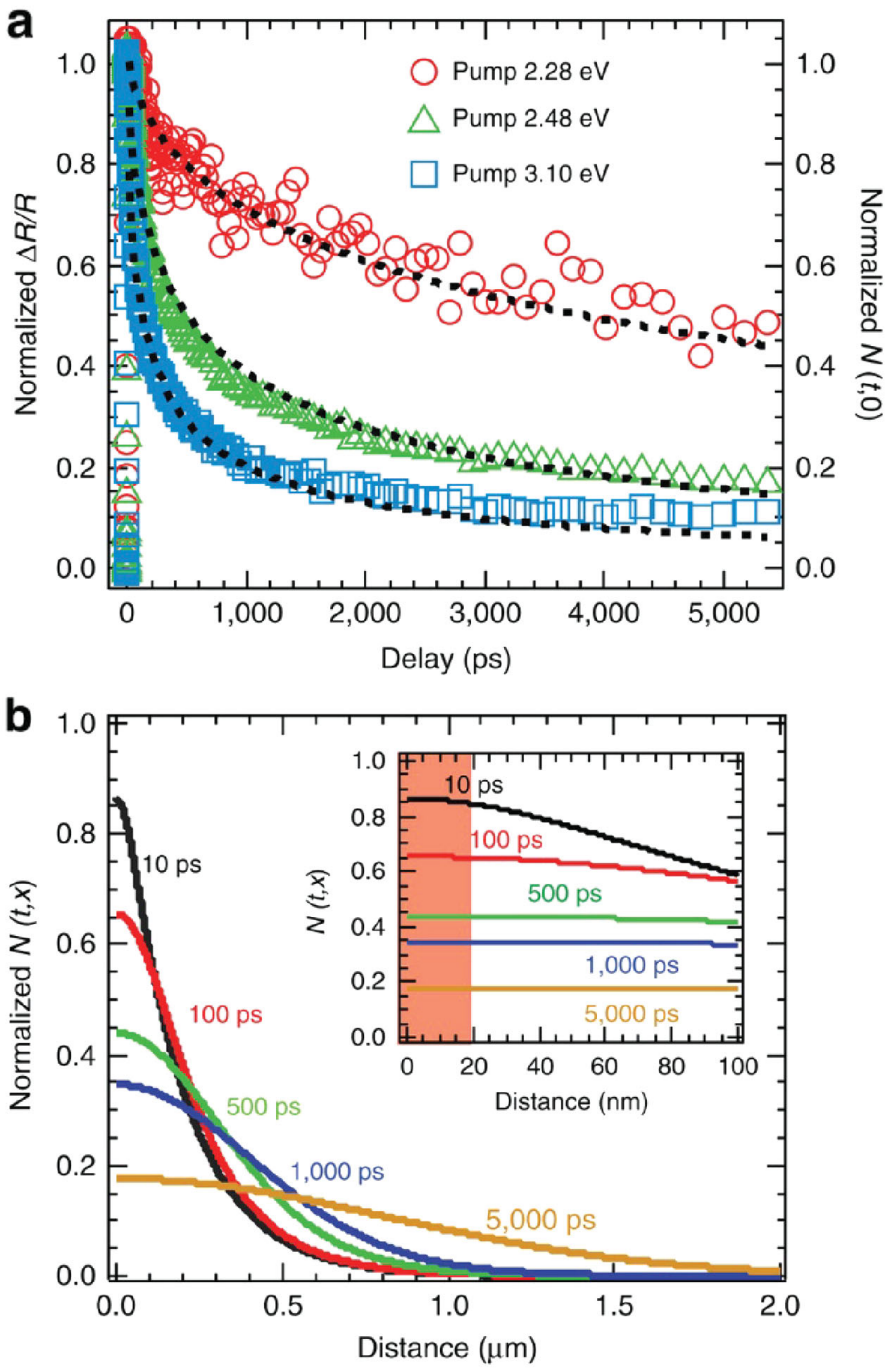
Excitation energy dependent TR kinetics and carrier density distribution profiles. a) The normalized TR kinetics recorded at 2.38 eV for three different pump energies. The normalized surface carrier density dynamics shows the same decay trend as the TR kinetics. The black dashed lines represent the global fitting based on the carrier diffusion model. b) Normalized carrier density distribution profiles for 2.48 eV pump at indicated delays in the single crystal. Inset: The distributions within 100 nm from the surface; the red shade represents the probe (at 2.38 eV) detection depth. a,b) Reproduced with permission.^130^ Copyright 2015, Nature Publishing Group.

Large‐size perovskite single crystals are an excellent platform to carry out foundamental studies of intrinsic photophysics due to the elimination of grain boundary scatterings.^126^, ^127^, ^128^, ^129^ For instance, surface recombination dynamics in CH_3_NH_3_PbBr_3_ single crystals were investigated by using broadband transient reflectance (TR) spectroscopy.^130^ In the TR measurement, reflectance (*R*) can be modulated by optical excitation of the perovskite single crystal while near the bandgap the relative reflectance change (Δ*R*/*R*) was recorded by a white‐light continuum. **Figure** **8** displays such excitation energy dependent TR kinetics and carrier density distribution profiles. Figure 8a shows a linear relationship between Δ*R*/*R* and the excitation energy, suggesting that TR kinetics followed the total carrier dynamics in the effective detecting region. One dimensional diffusion model was used to understand the relationship between carrier density and surface recombination. By a non‐linear global fitting routine and simultaneous calculation, the ambipolar mobility was estimated as 10.8 cm^2^ V^‐1^ s^‐1^ via the Einstein relation, which is much larger than that obtained for perovskite polycrystalline films. Moreover, the surface recombination velocity of CH_3_NH_3_PbBr_3_ single crystals was determined as 3.4 ± 0.1 × 10^3^ cm s^‐1^, which was 2−3 orders of magnitude lower than traditional semiconductors. In order to eliminate negative effects of such surface recombination, grain size of perovskite thin films should be larger than 30 mm. Figure 8b presents normalized carrier density distribution profiles for 2.48 eV pump in the single crystal. It was uncovered that the carrier density decreased near the surface and increased in the bulk, which could be attributed to the diffusion effect. Carrier distribution was nearly uniform in the effective detecting depth of 18 nm, confirming that the effect of carrier density on ∆*R*/*R* can be safely approximated by the surface density, as indicated form the insets. Furthermore, transient multi‐THz spectroscopy revealed a low binding energy of 17 meV and remarkably high carrier mobilities of 800 cm^2^ V ^‐1^ s^‐1^ on subpicosecond time scales in CH_3_NH_3_PbI_3_ single crystals.^131^

**Scheme 1:**
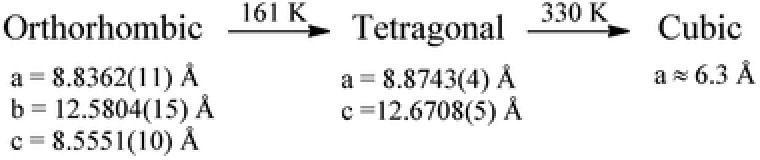
Phase transitions of CH_3_NH_3_PbI_3_ at different temperatures.

It is generally believed that CH_3_NH_3_PbI_3_ undergoes multiple phase transitions as a function of temperature,^1^ as shown in **Scheme** **1**.

In order to better understand these structures, a temperature variable ^1^H and ^13^C magic angle spinning nuclear magnetic resonance (MAS‐NMR) study of perovskite single crystals was conducted.^132^ The ^1^H and ^13^C NMR spectra showed that CH_3_NH_3_^+^ units underwent dynamic reorientation because the organic components tumbled in the perovskite cages formed by the PbI_6_ octahedral. In addition, only the amine end of the MA group interacted with the inorganic network. It is interesting to note that high‐temperature phase transition of CH_3_NH_3_PbI_3_ would lead to a large reduction in resistivity, which was desirable for high‐temperature applications. Recently, Grancini et al. revealed that the surface of CH_3_NH_3_PbI_3_ single crystals was structurally inhomogeneous.^133^ The edge surface exhibited a larger band gap and shorter carrier recombination dynamics than the center. This phenomenon was caused by the local distortion of crystal lattice at the crystal edges upon humidity exposure.

#### 3.1.2 2D Nanoplates

Aside from 3D large‐sized single crystals, 2D perovskites such as nanosheets, nanoplatelets and microdisks have recently been shown to exhibit high PL quantum yield.^134^, ^135^, ^136^ These 2D perovskites are promising candidates for a variety of applications in nanoelectronics, nanophotonics, and photovoltaics. Furthermore, these nanoscale building blocks can be empolyed in both fundamental studies.^137^, ^138^

For instance, Zhao et al. prepared MAPbI_2_Br nanosheets with a 1.8 eV band gap through a thermal decomposition process from a precursor containing PbI_2_, MABr and MACl.^64^ The planar solar cell based on the compact layer of MAPbI_2_Br nanosheets exhibited a PCE of ≈10%.

**Figure 9:**
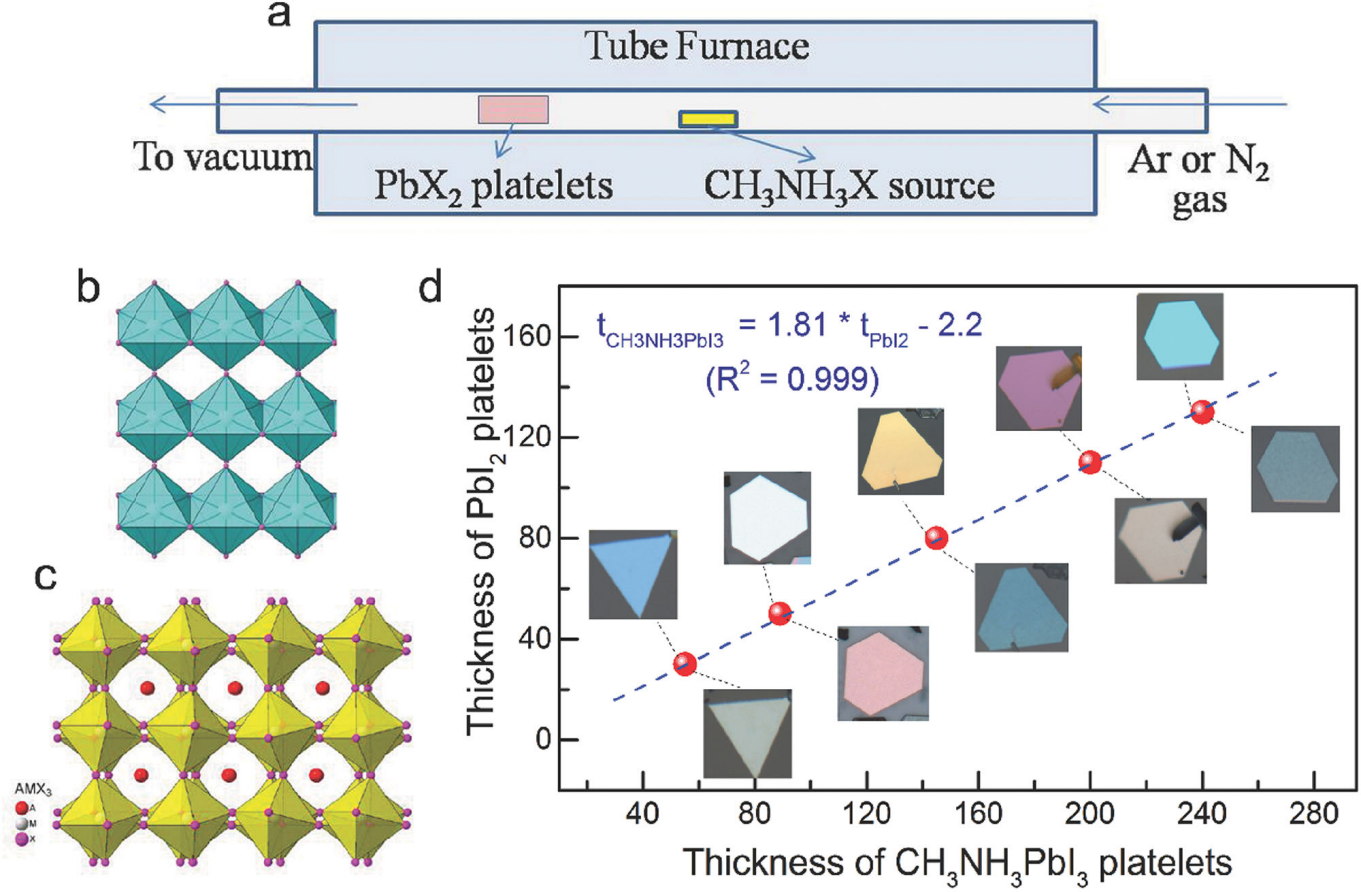
Conversion of lead halide nanoplatelets to perovskites by gas–solid heterophase reaction with methyl ammonium halide. a) Schematic of the synthesis setup using a home‐built vapor‐transport system. Crystal structure of b) lead halide and c) lead halide perovskite CH_3_NH_3_PbX_3_. d) Thickness of PbI_2_ platelets before (images above data line) and after being converted to CH_3_NH_3_PbI_3_ (images below data line). Note that the color of the PbI_2_ platelets changed corresponding to the change in thickness (as measured by AFM). a–d) Reproduced with permission.^139^ Copyright 2014, Wiley‐VCH.

As another example, the Xiong group successfully grew well‐defined polygonal CH_3_NH_3_Pb*X*_3_ (*X* = Cl, Br, I) nanoplatelets by chemical vapor method.^139^ As shown in **Figure** **9**a, Pb*X*_2_ platelets were first prepared on muscovite mica using van der Waals epitaxy in a vapor transport chemical deposition system. Next, these Pb*X*_2_ platelets were placed downstream in a quartz tube, which was in vacuum with an inert carrier gas such as nitrogen or argon. With the temperature of tube furnace increasing to 120 °C, MAX was vaporized to initiate a gas–solid hetero‐phase reaction with Pb*X*_2_ to convert into perovskites. Figure 9b,c shows the crystal structure of Pb*X*_2_ and CH_3_NH_3_Pb*X*_3_. Pb*X*_2_ forms a layered structure with each octahedron sharing two equatorial halide atoms with its neighbors in the same layer, and one axial halide atom with its neighbors from different layers. In contrast, CH_3_NH_3_Pb*X*_3_ adopts a 3D network structure because each octahedron shares only one halide atom with its neighbors either in the same layer or in a different layer. The lattice constant *c* ratio of the two compounds was about 1.81, which was originated from the insertion of a methyl ammonium group in the center of eight octahedrons and the relocation of the equatorial halide atoms in CH_3_NH_3_Pb*X*_3_. Interestingly, the thickness of PbI_2_ correlated with CH_3_NH_3_PbI_3_ platelets by a factor of 1.81 (Figure 9d), which was in good agreement with above mentioned lattice constant ratio along the *c* axis. This work offers a reliable method to control the thickness of perovskite platelets. Later, the same group readily applied these perovskite nanoplatelets to fabricate near‐infrared solid‐state lasers, which exhibited low thresholds and wide mode‐tunability.^140^

Recently, Liao et al. fabricated single‐crystalline CH_3_NH_3_PbBr_3_ square mirodisks (MDs) based microlasers by using a one‐step solution self‐assembly method.^141^ This approach was similiar to antisolvent vapor‐assisted crystallization method and used to produce large‐size single crystals.^115^ The acquired square MDs had smooth outer surfaces and sharp edges, and displayed an absorption peak at 535 nm and a emission peak at 545 nm. Their four side‐faces constituted a built‐in whispering‐gallery mode microresonator with a quality factor as high as ≈430. By partial replacement of Br with Cl, the lasing wavelength can be effectively tuned in the green‐light range from 525 to 557 nm.

In a more recent study, 2D MAPbBr_3_ nanoplatelets with nearly single unit cell thickness and submicron lateral dimensions were prepared from colloidal synthesis method.^142^ These 2D nanoplatelets exhibited a single and sharp excitonic absorption feature at 431 nm, which blue‐shifted by 0.5 eV from that of the 3D bulk perovskite phase. This large blue‐shift showed a clear evidence of quantum confinement in one dimension. Similar colloidal synthetic method was used to grow CsPbBr_3_ nanoplatelets, which exhibited narrow PL and strong excitionic absorption.^143^ In a very recent report, Jang et al. synthesized MAPbBr_3_ nanoplates using octylamine as the capping ligand.^144^ The composition of nanoplates was tunable by simple halide exchange reaction of MAPbBr_3_ with MACl and MAI in IPA. Photodetectors based on these MAPbBr_3−*x*_Cl*_x_* and MAPbBr_3−*x*_I*_x_* nanoplates were fabricated, in which mixed halide perovskites with I‐rich composition (*x* = 2) exhibited the highest photocurrents. Most recently, Yang et al. reported the solution growth of atomically thin, uniform, and square shaped 2D hybrid perovskites of (C_4_H_9_NH_3_)_2_PbBr_4_.^145^ Different than conventional 2D materials, (C_4_H_9_NH_3_)_2_PbBr_4_ sheet exhibited an unusual lattice constant expansion, which led to a slightly shifted band edge emission relative to the bulky counterpart. These 2D crystals also displayed high PL quantum efficiency and color tunability through halide substitution and thickness variation.

Of an interesting note, perovskite nanosheets were surprisingly obtained from electrospraying the precursor solution into a mixed bath of toluene (as antisolvent) and oleylamine (for intercalation).^146^ Recently, single crystals of CH_3_NH_3_PbI_3_ nanoplates with well‐defined facets were grown in the solution via a dissolution‐recrystallization path from PbI_2_ (or PbAc_2_) films coated on substrate.^147^ This method also yielded CH_3_NH_3_PbI_3_ nanowires. These 1D and 2D perovskite nanostructures displayed strong room‐temperature PL and long carrier lifetime. Moreover, surface photovoltage measurements indicated their n‐type characteristic.

#### 3.1.3 1D Nanowires

1D nanostructured semiconducting materials including fibers, wires, rods and tubes have manifested to exhibit excellent performance in nanoelectronics, photoelectronics, and data storages because of their anisotropic geometry and small size effect. It is thus anticipated that 1D organolead trihalide perovskites would show more intriguing features than their bulk counterparts by integrating various advantages of perovskites and 1D materials.

The 1D perovskite nanostructures can be attained by both physical and chemical methods. For example, CH_3_NH_3_PbI_3_ nanowires were prepared through a simple slip‐coating method, yielding a mean diameter of 50−400 nm and a length up to 10 μm.^148^ Such anisotropic perovskite nanowires outperformed nanoparticles in terms of charge transport under illumination. In a later study, Zhu and co‐workers synthesized MAPb*X*_3_ (*X* = I, Br) nanowires and nanorods by adding Pb*X*_2_ and MAX precursor solutions in polar solvents (e.g., CH_3_CN, GBL, DMF) to a nonpolar crashing solvents (e.g., toluene), assisted by a cation capping agent (e.g., *n*‐octylammonium).^149^ By varying the initial addtion rate and concentration of precursor as well as capping ligands, perovskite crytsals with different morphologies were also obtained from bulk, plates to dots.

Recently, 1D perovskite nanostructures began to appear in a wide range of applications. The Park group demonstrated the application of CH_3_NH_3_PbI_3_ nanowires for solar cells.^150^ By addition of a small quantity of DMF in the second step of two‐step spin‐coating procedure, CH_3_NH_3_PbI_3_ nanowires with the mean diameter of 100 nm were successfully grown. These nanowires had improved hole injection from perovskite to spiro‐MeOTAD and increased lateral conductivity compared to the 3D nanocuboid crystal, resulting in a PCE of 14.71%.

In addition, perovskite nanowires have been utilized in photodetectors. For instance, Zhang et al. synthesized porous CH_3_NH_3_PbBr_3_ nanowires through a self‐template directed reaction of Pb‐containing nanowires with HBr and CH_3_NH_3_Br in solution at room temperature.^151^ Photodetectors based on these porous nanowires exhibited high stability and sensitivity because of their unique porous 1D geometry. Later, Horváth et al. improved photodetector performance by constructing hybrid phototransistors, which consisted of slip‐coated CH_3_NH_3_PbI_3_ nanowires and CVD‐grown monolayered graphenes.^152^ These devices displayed remarkably high responsivities of 2.6 × 10^6^ A W^−1^ in the visible range.

**Figure 10:**
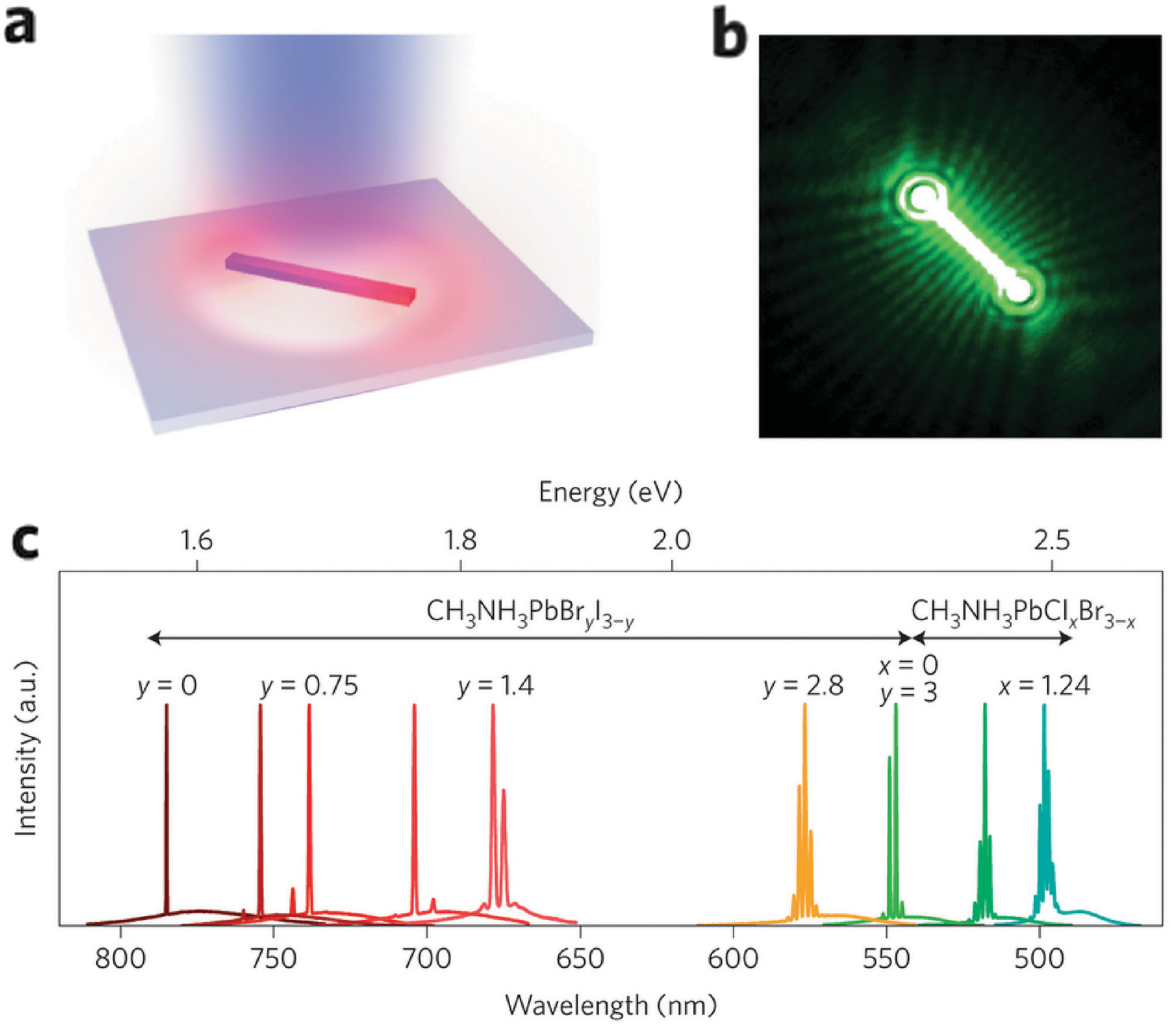
Lasing in hybrid organic–inorganic metal halide perovskite nanowires. a) Illustration of an optically pumped perovskite nanowire on a Si substrate with 300‐nm‐thick SiO_2_. b) Optical image of a 13.6‐μm‐long CH_3_NH_3_PbBr_3_ nanowire showing lasing emission. c) Lasing spectra of various alloy compositions of mixed lead halide perovskite nanowires demonstrating widely tunable laser emission at room temperature. a–c) Reproduced with permission.^11^ Copyright 2015, Nature Publishing Group.

Moreover, perovskite nanowires were introduced as ideal building blocks for laser applications. For example, Zhu and colleagues fabricated perovskite nanowire lasers with very low lasing threshold of 220 nJ cm^‐2^, high quality factor of 3,600, near‐unity quantum yield (QY) and importantly broad tunability covering the near‐infrared to visible wavelength region.^11^, ^153^ In their work, well‐dispersed perovskite nanowires were grown by contacting a PbAc_2_ thin film with a high concentration of CH_3_NH_3_X solution in IPA. These nanowires were transferred to Si/SiO_2_ substrate by a simple dry contact process, as shown in **Figure** **10**a, and then optically pumped by a 402 nm pulsed laser beam under a far‐field epi‐fluorescence microscope at room temperature in a dry N_2_ atmosphere. As a result, single‐crystal CH_3_NH_3_PbBr_3_ nanowires exhibited efficient lasing in the green spectral region (Figure 10b). Such exceptional lasing performance is believed to originate from long carrier lifetime and low non‐radiative recombination rates of rectangular perovskite nanowires in cross‐section. Simply by changing the ratio of methylammonium iodide and bromide or bromide and chloride in the precursor solution, MAPbBr*_x_*I_3‐*x*_ and MAPbCl*_x_*Br_3‐*x*_ nanowires with various stoichiometries can be obtained. Importantly, this enabled broad wavelength tunability covering the near‐infrared to visible region (Figure 10c). As another example, Xing et al. prepared free‐standing perovskite nanowires through vapor‐phase synthesis.^154^ These perovskite nanowires constituted a built‐in Fabry−Pérot microresonator, leading to low‐threshold optically pumped lasing with a near‐infrared wavelength of 777 nm and a quality factor as high as 405.

More recently, 1D perovskite nanorods found applications in light‐emitting diode (LED).^155^ The growth of CH_3_NH_3_PbBr_3_ nanorod array was fulfilled by placing PbAc_2_ thin film into a solution of CH_3_NH_3_Br in IPA, similar to Zhu's report.^11^ These CH_3_NH_3_PbBr_3_ nanorods were directly converted into CH_3_NH_3_PbI_3_ nanorods via anion exchange. Both nanorod arrays exhibited the electroluminescence at 533 and 782 nm, respectively.

#### 3.1.4 0D Quantum Dots

Semiconductor quantum dots (QDs) have received exponentially increasing attention due to their unique opto‐electronic properties, such as size tunable absorption and bandgap, high optical extinction coefficient and multiple exciton generation characteristics. Perovskites QDs are expected to exhibit interesting nanoscale excitonic properties and also hold other potential applications in transistors, diode lasers, LEDs, and fluorescent biomedical imaging.

The photovoltaic applications of CH_3_NH_3_Pb*X*_3_ perovskite nanoparticles were first explored by Miyasaka et al. in 2009.^5^ Perovskite nanoparticles were deposited on the surface of mesoporous TiO_2_ by a self‐organization process. Later, the Park group fabricated 6.54% efficient QD‐sensitized solar cell with 2−3 nm sized CH_3_NH_3_PbI_3_ nanocrystals adhered on nanocrystalline TiO_2_ surface.^156^ Aside from the above‐mentioned mesoporous metal oxides confined perovskite particles, the Pérez‐Prieto group obtained free and nanometer‐sized CH_3_NH_3_PbBr_3_ nanoparticles as stable colloidal solutions.^157^ The key is to use an ammonium bromide with a medium‐sized chain in the reaction of CH_3_NH_3_Br with PbBr_2_ in the presence of oleic acid and octadecene. The ammonium ions acted as capping ligands, which limited the growth of CH_3_NH_3_PbBr_3_ toward three dimensions. Both the colloidal solution and thin film exhibited a high QY of ca. 20% with a narrow emission band. The same group furthur enhanced the QY to 83% by fine‐tuning of the molar ratio of precursors and ligands.^158^

**Figure 11:**
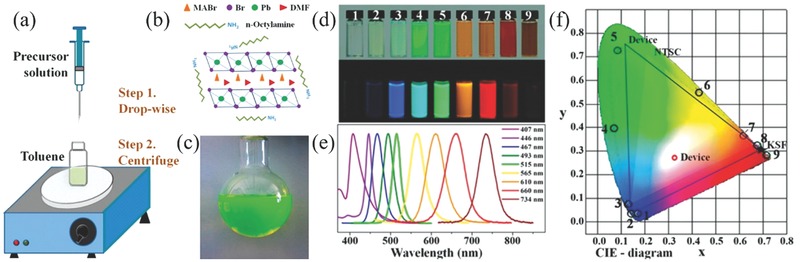
a) Schematic illustration of the reaction system and process for LARP technique. b) Schematic illustration of starting materials in the precursor solution. c) Typical optical image of colloidal CH_3_NH_3_PbBr_3_ solution. d) Optical images of CH_3_NH_3_PbX_3_ QDs (No. 1–9) under ambient light and a 365 nm UV lamp. e) PL emission spectra of CH_3_NH_3_PbX_3_ QDs. f) CIE color coordinates corresponding to the CH_3_NH_3_PbX_3_ QDs (No. 1–9, black circle), pc‐WLED devices (blue lines), and NTSC standard (bright area). a–f) Adapted with permission.^159^ Copyright 2015, American Chemical Society.

Recently, Zhang et al. developed a ligand‐assisted reprecipitation (LARP) technique to produce brightly luminescent and color‐tunable colloidal CH_3_NH_3_Pb*X*_3_ (*X* = Br, I, Cl) QDs with QYs up to 50−70%.^159^] **Figure** **11** schematically illustrates such a LARP fabrication process. Figure 11a exemplifies a typical synthesis of CH_3_NH_3_PbBr_3_ QDs, in which a precursor solution was prepared by mixing PbBr_2_, CH_3_NH_3_Br, *n*‐octylamine, and oleic acid in DMF. Free‐standing layered precursors were then formed in DMF (Figure 11b). Subsequently, a fixed amount of precursor solution was dropped into toluene under vigorous stirring, yielding a yellow‐green colloidal solution of small‐sized nanoparticles (Figure 11c). By varying the composition of cations, the absorption and emission spectra were finely tuned from 407 to 734 nm (Figure 11d,e). Figure 11f is the commission international de L'Eclarage (CIE) chromaticity diagram of these QDs, which exhibited high color saturation due to their relative narrow emissions. Finally, wide‐color gamut white‐light‐emitting diodes were fabricated based on green emissive CH_3_NH_3_PbBr_3_ QDs and red emissive K_2_SiF_6_:Mn_4_þ as color converters.

In another study, CH_3_NH_3_PbBr_3_ QDs with impressively high absolute QY of 74−93% were synthesized by a facile ligand‐assisted reprecipitation technique.^160^ Such high QY was attributed to both short radiative lifetime of 13−27 ns and nonradiative lifetime of 100 ns. Apart from CH_3_NH_3_PbX_3_ QDs, CsPbX_3_ QDs have been synthesised, which exhibited superior properties such as narrow full widths at half−maximum, remarkably high PL QYs (approximately ≥ 70 %) and tunable broad spectral window.^161^, ^162^, ^163^, ^164^ Furthermore, CH_3_NH_3_PbI_3_ perovskites were successfully used as stable capping ligands for a range of CQDs such as PbS, CdS, InP and CdSe via solution ligand‐exchange reactions, resulting in efficient electronic passivation for highly luminescent CQDs.^165^ More recently, PbS‐in‐perovskite solids were fabricated through in‐situ epitaxial growth process in solution phase, leading to superior carrier transport between perovskites and PbS.^166^ Most recently, stable CH_3_NH_3_PbI_3_ capped PbS QDs were obtained though a solid‐state ligand exchange method, which were further used as the absorber in solar cells.^167^

### 3.2 Growth Mechanisms

Understanding the crystal growth mechanism is of great importance for optimization of synthetic methods and further applications. Typically, crystal growth in solution can be divided into three basic types: precipitation from supersaturated solution, in situ transformation, and dissolution−crystallization.^168^

**Figure 12:**
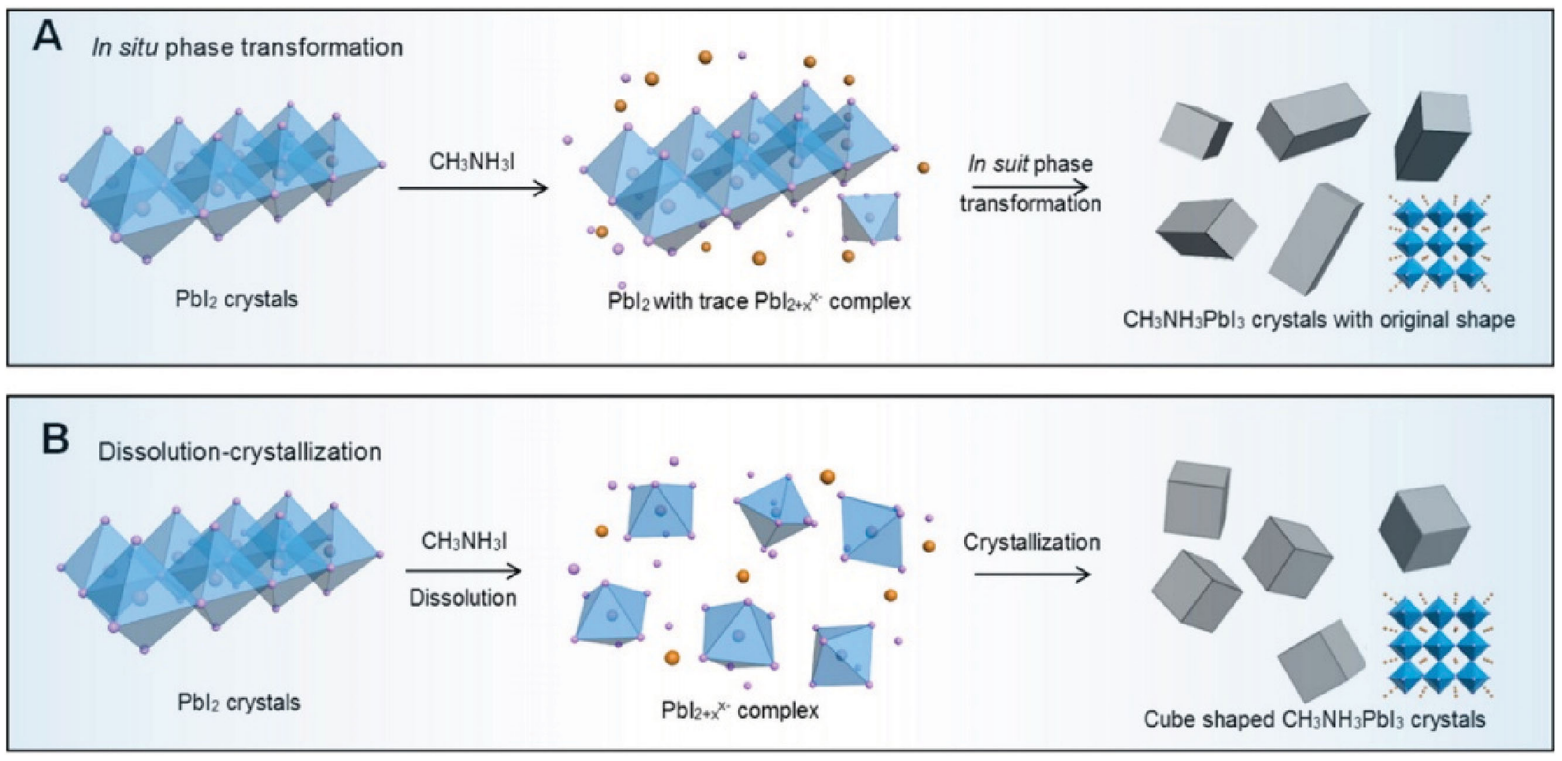
Schematic illustration of the plausible formation mechanisms of A) in situ transformation and B) dissolution−crystallization of CH_3_NH_3_PbI_3_ crystals via sequential reaction route. The insets in the right images are the atomic structure of CH_3_NH_3_PbI_3_ crystals. A,B) Reproduced with permission.^169^ Copyright 2014, American Chemical Society.

For the sequential reaction route, crystal growth is dominated by in‐situ transformation or dissolution−crystallization mechanisms. As displayed in **Figure** **12**, PbI_2_ adopts layered structure with a hexagonally close‐packed Pb plane sandwiched between two layers of iodide ions. Yang et al. found that the kinetics of such crystal growth process strongly relied on the CH_3_NH_3_I concentration.^169^ At a low CH_3_NH_3_I concentration (<16.5 mM), the growth process followed in‐situ transformation mechanism. Only a traceable amount of PbI_2_ was dissolved in solvent. As a result, the CH_3_NH_3_PbI_3_ crystals maintained inorganic lead framework. When the CH_3_NH_3_I concentration was over 26.7 mM, the dissolution−crystallization mechanism dominated the reaction. PbI_2_ was fully coordinated with iodine ions to form PbI_2+*x*_^x‐^ in an iodine rich environment, by Equation (2):^170^

(2) $${\mathrm{PbI}}_{2}+{\mathrm{xI}}^{-}{\mathrm{PbI}}_{\mathrm{2+x}}^{\mathrm{x‐}}\left(x=1,2\right)$$

Then lead complex further acted as as building blocks to recrystallize into a thermodynamically favored morphology in the presence of ammonium cations via Equation (3):

(3) $${\mathrm{CH}}_{3}{\mathrm{NH}}_{3}+{\mathrm{PbI}}_{\mathrm{2+x}}^{\mathrm{x‐}}{\mathrm{CH}}_{3}{\mathrm{NH}}_{3}{\mathrm{PbI}}_{3}+\left(x-1\right)I^{‐}\;\;\left(x=1,2\right)$$

For the medium CH_3_NH_3_I concentration, both mechanisms worked together. From above formation mechanism, the morphology of CH_3_NH_3_PbI_3_ functional crystals can be elaborately tailored by controlling the PbI_2_ morphology and CH_3_NH_3_I concentration.

Later, Ostwald ripening mechanism was found to exist in one‐pot solvothermal method to prepare cuboid shaped CH_3_NH_3_PbI_3_ single crystals.^171^ Small crystals were dissolved and re‐deposited onto the surface of larger crystals. Dissolution phenomenon from specific facets was unraveled, under high temperature and long time of reactions, as a dynamic process of balancing dissolution and recrystallization.

Recently, Zheng et al. found that perovskite crystal growth in solid state followed a Volmer−Weber growth mechanism during hot‐casting.^96^ Island shaped grains were first formed and subsequently integrated into dense perovskite films. The process was determined by the synergetic effect of thermal energy and force centrifugal field.

## 4 Conclusions and Outlook

In summary, we have reviewed recent developments of structure and growth of organic–inorganic halide perovskites towards high‐performance electronic devices. Two major forms of perovskites are under discussion, polycrystalline films and single crystals. Film formation process of perovskites plays a paramount role in determining their final photovoltaic and light‐emitting diode performance. Perovskite films can be prepared by a variety of techniques, mainly including one‐step solution processing, two‐step sequential deposition, vapor deposition and vapor assisted solution processing. In addition, other viable deposition methods such as CVD, spray‐coating, blade coating, and slot die have been successfully applied to fabricate high‐quality perovskite films, paving the way for low‐cost mass production of perovskite‐based electronic devices.

Perovskite film morphology is of critical importance for device performance, which can be optimized by various strategies such as the use of additives, thermal annealing, solvent annealing, atmospheric control, and solvent engineering. The key is to entail a fine control over crystallization and growth of perovskites, aiming to produce uniform films with full surface coverage, large crystal size and even good stability. It is worth noting that chloride additives including HCl, NH_4_Cl, MACl, FACl, TPPCl and 4‐ABPACl were found to play a favorable role in the morphological evolution of perovskite thin films, which is very likely due to the chelation between Cl^−^ anions and Pb^2+^ cations. Moreover, when selecting the additives, their volatility should be considered. Interestingly, DIO and CN, frequently used additives for OPVs, can be readily employed to regulate the crystallization of perovskites. Solvent engineering and hot‐casting techniques were demonstrated to benefit the formation of large crystal domains. It is thus believed that a combination of improved processing protocols and proper morphological control avenues would lead to significant breakthrough of perovskite based solar cells in the furture.

Perovskites single crystals are receiving increasing attention because of their remarkably lower trap densities, higher charge mobilities, longer carrier diffusion lengths and better stability than their polycrystalline thin film counterparts. It has been found that shape and size of perovskite single crystals are intimately correlated to their optoelectronic properties. We have then summarized shape evolution of perovskites single crystals from 3D large sized single crystals, 2D nanoplates, 1D nanowires to 0D quantum dots. Large‐sized single crystals are usually prepared by antisolvent vapor‐assisted crystallization, seed solution‐growth method, cooling‐induced crystallization and solvothermal growth methods. Intriguingly, two‐inch‐sized single crystals were successful grown from a repeated seed‐solution growth method. In addition, inverse temperature crystallization demonstrated a rapid crystal growth approach. Of particular importance, they are ideal platforms for foundamental studies due to the absence of grain boundries, especially in the context of charge recombination dynamics and carrier mobility measurement. Furthermore, perovskites nanoplatelets, microdisks, nanowires and nanodots can grow via the competition between in‐situ transformation and dissolution−crystallization mechanisms by controlling the PbI_2_ morphology and CH_3_NH_3_I concentration. Besides, the use of caping ligands and crashing solvents facilitate to tailor the shape of perovskite single crystals. Importantly, low dimensional perovskites have exhibited high PL quantum yields, holding huge promise for high‐performance optoelectronic applications such lasers, photodetectors, and LEDs.

Emerging applications of these free‐standing perovskite single crystals are mostly based on the constrction of one‐crystal devices, which is however of high technical difficulty. Another prime obstacle limiting their further applications is the need of forming ordered arrangement and desirable contact on flat substrates. Nevertheless, one latest study showed great promise by employing antisolvent vapor‐assisted crystallization technique to fabricate planar‐integrated single‐crystalline perovskites, which exhibited superior photodetector performance.^172^ Of course, more effective strategies such as in‐situ growth methods are urgently needed to warrant the optoelectronic integration of single crystals.

## References

[1] H.‐S. Kim , S. H. Im , N.‐G. Park , J. Phys. Chem. C 2014, 118, 5615.

[2] S. Kazim , M. K. Nazeeruddin , M. Grätzel , S. Ahmad , Angew. Chem. Int. Ed. 2014, 53, 2812. 10.1002/anie.201308719 24519832

[3] Y. Rong , L. Liu , A. Mei , X. Li , H. Han , Adv. Energy Mater. 2015, 5, 1501066.

[4] J. Berry , T. Buonassisi , D. A. Egger , G. Hodes , L. Kronik , Y.‐L. Loo , I. Lubomirsky , S. R. Marder , Y. Mastai , J. S. Miller , D. B. Mitzi , Y. Paz , A. M. Rappe , I. Riess , B. Rybtchinski , O. Stafsudd , V. Stevanovic , M. F. Toney , D. Zitoun , A. Kahn , D. Ginley , D. Cahen , Adv. Mater. 2015, 27, 5102. 26223962 10.1002/adma.201502294

[5] A. Kojima , K. Teshima , Y. Shirai , T. Miyasaka , J. Am. Chem. Soc. 2009, 131, 6050. 19366264 10.1021/ja809598r

[6] H.‐S. Kim , C.‐R. Lee , J.‐H. Im , K.‐B. Lee , T. Moehl , A. Marchioro , S.‐J. Moon , R. Humphry‐Baker , J.‐H. Yum , J. E. Moser , M. Grätzel , N.‐G. Park , Sci. Rep. 2012, 2, 591. 22912919 10.1038/srep00591 PMC3423636

[7] M. M. Lee , J. Teuscher , T. Miyasaka , T. N. Murakami , H. J. Snaith , Science 2012, 338, 643. 23042296 10.1126/science.1228604

[8] M. Liu , M. B. Johnston , H. J. Snaith , Nature 2013, 501, 395. 24025775 10.1038/nature12509

[9] Best Research Cell Efficiencies: http://www.nrel.gov/ncpv/images/efficiency_chart.jpg (accessed: January 2016).

[10] F. Laquai , Nat. Mater. 2014, 13, 429. 24751766 10.1038/nmat3953

[11] H. Zhu , Y. Fu , F. Meng , X. Wu , Z. Gong , Q. Ding , M. V. Gustafsson , M. T. Trinh , S. Jin , X.‐Y. Zhu , Nat. Mater. 2015, 14, 636. 25849532 10.1038/nmat4271

[12] S. Yakunin , M. Sytnyk , D. Kriegner , S. Shrestha , M. Richter , G. J. Matt , H. Azimi , C. J. Brabec , J. Stangl , M. V. Kovalenko , W. Heiss , Nat. Photonics 2015, 9, 444. 10.1038/nphoton.2015.82 PMC5444515 28553368

[13] M. He , Y. Chen , H. Liu , J. Wang , X. Fang , Z. Liang , Chem. Commun. 2015, 51, 9659. 10.1039/c5cc02282g 25977949

[14] Z.‐K. Tan , R. S. Moghaddam , M. L. Lai , P. Docampo , R. Higler , F. Deschler , M. Price , A. Sadhanala , L. Pazos , D. Credgington , F. Hanusch , T. Bein , H. J. Snaith , R. H. Friend , Nat. Nanotechnol. 2014, 9, 687. 25086602 10.1038/nnano.2014.149

[15] R. L. Z. Hoye , M. R. Chua , K. P. Musselman , G. Li , M.‐L. Lai , Z.‐K. Tan , N. C. Greenham , J. L. MacManus‐Driscoll , R. H. Friend , D. Credgington , Adv. Mater. 2015, 27, 1414. 25573086 10.1002/adma.201405044 PMC4515082

[16] Y.‐H. Kim , H. Cho , J. H. Heo , T.‐S. Kim , N. Myoung , C.‐L. Lee , S. H. Im , T.‐W. Lee , Adv. Mater. 2015, 27, 1248. 25420784 10.1002/adma.201403751

[17] M. Grätzel , Nat. Mater. 2014, 13, 838. 25141800 10.1038/nmat4065

[18] T. C. Sum , N. Mathews , Energy Environ. Sci. 2014, 7, 2518.

[19] A. S. Bhalla , R. Y. Guo , R. Roy , Mater. Res. Innovations 2000, 4, 3.

[20] C. Li , X. Lu , W. Ding , L. Feng , Y. Gao , Z. Guo , Acta Crystallogr 2008, B64, 702. 10.1107/S0108768108032734 19029699

[21] J. H. Noh , S. H. Im , J. H. Heo , T. N. Mandal , S. I. Seok , Nano Lett. 2013, 13, 1764. 23517331 10.1021/nl400349b

[22] K. Zheng , Q. Zhu , M. Abdellah , M. E. Messing , W. Zhang , A. Generalov , Y. Niu , L. Ribaud , S. E. Canton , T. Pullerits , J. Phys. Chem. Lett. 2015, 6, 2969. 26267190 10.1021/acs.jpclett.5b01252

[23] S. D. Stranks , G. E. Eperon , G. Grancini , C. Menelaou , M. J. P. Alcocer , T. Leijtens , L. M. Herz , A. Petrozza , H. J. Snaith , Science 2013, 342, 341. 24136964 10.1126/science.1243982

[24] G. Xing , N. Mathews , S. Sun , S. S. Lim , Y. M. Lam , M. Grätzel , S. Mhaisalkar , T. C. Sum , Science 2013, 342, 344. 24136965 10.1126/science.1243167

[25] T. Leijtens , S. D. Stranks , G. E. Eperon , R. Lindblad , E. M. J. Johansson , I. J. McPherson , H. Rensmo , J. M. Ball , M. M. Lee , H. J. Snaith , ACS Nano 2014, 8, 7147. 24949826 10.1021/nn502115k

[26] Y. Chen , J. Peng , D. Su , X. Chen , Z. Liang , ACS Appl. Mater. Interfaces 2015, 7, 4471. 25695862 10.1021/acsami.5b00077

[27] C. Wehrenfennig , G. E. Eperon , M. B. Johnston , H. J. Snaith , L. M. Herz , Adv. Mater. 2014, 26, 1584. 24757716 10.1002/adma.201305172 PMC4722848

[28] Y. Zhao , K. Zhu , J. Phys. Chem. Lett. 2014, 5, 4175. 26278951 10.1021/jz501983v

[29] G. E. Eperon , V. M. Burlakov , P. Docampo , A. Goriely , H. J. Snaith , Adv. Funct. Mater. 2014, 24, 151.

[30] J. Huang , Y. Shao , Q. Dong , J. Phys. Chem. Lett. 2015, 6, 3218.

[31] S. D. Stranks , P. K. Nayak , W. Zhang , T. Stergiopoulos , H. J. Snaith , Angew. Chem. Int. Ed. 2015, 54, 3240. 10.1002/anie.201410214 25663077

[32] T.‐B. Song , Q. Chen , H. Zhou , C. Jiang , H.‐H. Wang , Y. M. Yang , Y. Liu , J. You , Y. Yang , J. Mater. Chem. A 2015, 3, 9032.

[33] Q. Chen , N. D. Marco , Y. M. Yang , T.‐B. Song , C.‐C. Chen , H. Zhao , Z. Hong , H. Zhou , Y. Yang , Nano Today 2015, 10, 355.

[34] J.‐H. Im , I.‐H. Jang , N. Pellet , M. Grätzel , N.‐G. Park , Nat. Nanotechnol. 2014, 9, 927. 25173829 10.1038/nnano.2014.181

[35] Q. Chen , H. Zhou , Z. Hong , S. Luo , H.‐S. Duan , H.‐H. Wang , Y. Liu , G. Li , Y. Yang , J. Am. Chem. Soc. 2014, 136, 622. 24359486 10.1021/ja411509g

[36] M. R. Leyden , L. K. Ono , S. R. Raga , Y. Kato , S. Wang , Y. Qi , J. Mater. Chem. A 2014, 2, 18742.

[37] S. Gamliel , A. Dymshits , S. Aharon , E. Terkieltaub , L. Etgar , J. Phys. Chem. C 2015, 119, 19722.

[38] Y. Deng , E. Peng , Y. Shao , Z. Xiao , Q. Dong , J. Huang , Energy Environ. Sci. 2015, 8, 1544.

[39] K. Hwang , Y.‐S. Jung , Y.‐J. Heo , F. H. Scholes , S. E. Watkins , J. Subbiah , D. J. Jones , D.‐Y. Kim , D. Vak , Adv. Mater. 2015, 27, 1241. 25581092 10.1002/adma.201404598

[40] J. M. Ball , M. M. Lee , A. Hey , H. J. Snaith , Energy Environ. Sci. 2013, 6, 1739.

[41] H. Zhou , Q. Chen , G. Li , S. Luo , T.‐b. Song , H.‐S. Duan , Z. Hong , J. You , Y. Liu , Y. Yang , Science 2014, 345, 542. 25082698 10.1126/science.1254050

[42] K. Wojciechowski , M. Saliba , T. Leijtens , A. Abate , H. J. Snaith , Energy Environ. Sci. 2014, 7, 1142.

[43] J. H. Heo , H. J. Han , D. Kim , T. K. Ahn , S. H. Im , Energy Environ. Sci. 2015, 8, 1602.

[44] B. Susrutha , L. Giribabu , S. P. Singh , Chem. Commun. 2015, 51, 14696. 10.1039/c5cc03666f 26198773

[45] B. J. Kim , D. H. Kim , Y.‐Y. Lee , H.‐W. Shin , G. S. Han , J. S. Hong , K. Mahmood , T. K. Ahn , Y.‐C. Joo , K. S. Hong , N.‐G. Park , S. Lee , H. S. Jung , Energy Environ. Sci. 2015, 8, 916.

[46] X. Hu , X. Zhang , L. Liang , J. Bao , S. Li , W. Yang , Y. Xie , Adv. Funct. Mater. 2014, 24, 737.

[47] J. Burschka , N. Pellet , S.‐J. Moon , R. Humphry‐Baker , P. Gao , M. K. Nazeeruddin , M. Grätzel , Nature 2013, 499, 316. 23842493 10.1038/nature12340

[48] Z. Xiao , C. Bi , Y. Shao , Q. Dong , Q. Wang , Y. Yuan , C. Wang , Y. Gao , J. Huang , Energy Environ. Sci. 2014, 7, 2619.

[49] C.‐H. Chiang , Z.‐L. Tseng , C.‐G. Wu , J. Mater. Chem. A 2014, 2, 15897.

[50] Y. Ma , L. Zheng , Y.‐H. Chung , S. Chu , L. Xiao , Z. Chen , S. Wang , B. Qu , Q. Gong , Z. Wu , X. Houc , Chem. Commun. 2014, 50, 12458. 10.1039/c4cc01962h 24852764

[51] Y. Xu , L. Zhu , J. Shi , S. Lv , X. Xu , J. Xiao , J. Dong , H. Wu , Y. Luo , D. Li , Q. Meng , ACS Appl. Mater. Interfaces 2015, 7, 2242. 25587643 10.1021/am5057807

[52] M. Jiang , J. Wu , F. Lan , Q. Tao , D. Gao , G. Li , J. Mater. Chem. A 2015, 3, 963.

[53] C. Bi , Y. Yuan , Y. Fang , J. Huang , Adv. Energy Mater. 2014, 5, 1401616.

[54] Y. Chen , B. Li , W. Huang , D. Gao , Z. Liang , Chem. Commun. 2015, 51, 11997. 10.1039/c5cc03615a 26120826

[55] M. M. Tavakoli , L. Gu , Y. Gao , C. Reckmeier , J. He , A. L. Rogach , Y. Yao , Z. Fan , Sci. Rep. 2015, 5, 14083. 26392200 10.1038/srep14083 PMC4585726

[56] M. R. Leyden , L. K. Ono , S. R. Raga , Y. Kato , S. Wang , Y. Qi , J. Mater. Chem. A 2015, 3, 16097.

[57] D. J. Lewis , P. O'Brien , Chem. Commun. 2014, 56, 6319. 10.1039/c4cc02592j 24799177

[58] D. S. Bhachu , D. O. Scanlon , E. J. Saban , H. Bronstein , I. P. Parkin , C. J. Carmalt , R. G. Palgrave , J. Mater. Chem. A 2015, 3, 9071.

[59] P. Luo , Z. Liu , W. Xia , C. Yuan , J. Cheng , Y. Lu , ACS Appl. Mater. Interfaces 2015, 7, 2708. 25581720 10.1021/am5077588

[60] P. Luo , Z. Liu , W. Xia , C. Yuan , J. Cheng , Y. Lu , J. Mater. Chem. A 2015, 3, 12443.

[61] A. T. Barrows , A. J. Pearson , C. K. Kwak , A. D. F. Dunbar , A. R. Buckley , D. G. Lidzey , Energy Environ. Sci. 2014, 7, 2944.

[62] S. Das , B. Yang , G. Gu , P. C. Joshi , I. N. Ivanov , C. M. Rouleau , T. Aytug , D. B. Geohegan , K. Xiao , ACS Photonics 2015, 2, 680.

[63] Y. Zhao , K. Zhu , J. Phys. Chem. C 2014, 118, 9412.

[64] Y. Zhao , K. Zhu , J. Am. Chem. Soc. 2014, 136, 12241. 25118565 10.1021/ja5071398

[65] Y. Chen , Y. Zhao , Z. Liang , Chem. Mater. 2015, 27, 1448.

[66] Y. Chen , Y. Zhao , Z. Liang , J. Mater. Chem. A 2015, 3, 9137.

[67] J. He , T. Chen , J. Mater. Chem. A 2015, 3, 18514.

[68] Z. Wang , Y. Zhou , S. Pang , Z. Xiao , J. Zhang , W. Chai , H. Xu , Z. Liu , N. P. Padture , G. Cui , Chem. Mater. 2015, 27, 7149.

[69] P.‐W. Liang , C.‐Y. Liao , C.‐C. Chueh , F. Zuo , S. T. Williams , X.‐K. Xin , J. Lin , A. K.‐Y. Jen , Adv. Mater. 2014, 26, 3748. 24634141 10.1002/adma.201400231

[70] C.‐C. Chueh , C.‐Y. Liao , F. Zuo , S. T. Williams , P.‐W. Liang , A. K.‐Y. Jen , J. Mater. Chem. A 2015, 3, 9058.

[71] X. Song , W. Wang , P. Sun , W. Ma , Z.‐K. Chen , Appl. Phys. Lett. 2015, 106, 033901.

[72] C. Sun , Q. Xue , Z. Hu , Z. Chen , F. Huang , H.‐L. Yip , Y. Cao , Small 2015, 27, 3344. 10.1002/smll.201403344 25682920

[73] X. Li , M. I. Dar , C. Yi , J. Luo , M. Tschumi , S. M. Zakeeruddin , M. K. Nazeeruddin , H. Han , M. Grätzel , Nat. Chem. 2015, 7, 703. 26291941 10.1038/nchem.2324

[74] H.‐L. Hsu , C.‐C. Chang , C.‐P. Chen , B.‐H. Jiang , R.‐J. Jeng , C.‐H. Cheng , J. Mater. Chem. A 2015, 3, 9271.

[75] X. Gong , M. Li , X.‐B. Shi , H. Ma , Z.‐K. Wang , L.‐S. Liao , Adv. Funct. Mater. 2015, 25, 6671.

[76] B. Conings , A. Babayigit , T. Vangerven , J. D'Haen , J. Manca , H.‐G. Boyena , J. Mater. Chem. A 2015, 3, 19123.

[77] C.‐Y. Chang , C.‐Y. Chu , Y.‐C. Huang , C.‐W. Huang , S.‐Y. Chang , C.‐A. Chen , C.‐Y. Chao , W.‐F. Su , ACS Appl. Mater. Interfaces 2015, 7, 4955. 25679316 10.1021/acsami.5b00052

[78] G. E. Eperon , S. D. Stranks , C. Menelaou , M. B. Johnston , L. M. Herz , H. J. Snaith , Energy Environ. Sci. 2014, 7, 982.

[79] G. E. Eperon , G. M. Paterno , R. J. Sutton , A. Zampetti , A. A. Haghighirad , F. Cacialli , H. J. Snaith , J. Mater. Chem. A 2015, 3, 19688.

[80] J. H. Heo , D. H. Song , H. J. Han , S. Y. Kim , J. H. Kim , D. Kim , H. W. Shin , T. K. Ahn , C. Wolf , T.‐W. Lee , S. H. Im , Adv. Mater. 2015, 27, 3424. 25914242 10.1002/adma.201500048

[81] L. Yang , J. Wang , W. W.‐F. Leung , ACS Appl. Mater. Interfaces 2015, 7, 14614. 26108296 10.1021/acsami.5b01049

[82] G. Li , T. Zhang , Y. Zhao , J. Mater. Chem. A 2015, 3, 19674.

[83] C.‐G. Wu , C.‐H. Chiang , Z.‐L. Tseng , M. K. Nazeeruddin , A. Hagfeldt , M. Grätzel , Energy Environ. Sci. 2015, 8, 2725.

[84] Y. Xie , F. Shao , Y. Wang , T. Xu , D. Wang , F. Huang , ACS Appl. Mater. Interfaces 2015, 7, 12937. 26009927 10.1021/acsami.5b02705

[85] T. Zhang , M. Yang , Y. Zhao , K. Zhu , Nano Lett. 2015, 15, 3959. 25996160 10.1021/acs.nanolett.5b00843

[86] Y. Zhao , K. Zhu , J. Mater. Chem. A 2015, 3, 9086.

[87] K. W. Tan , D. T. Moore , M. Saliba , H. Sai , L. A. Estroff , T. Hanrath , H. J. Snaith , U. Wiesner , ACS Nano 2014, 8, 4730. 24684494 10.1021/nn500526t PMC4046796

[88] S. Aharon , A. Dymshits , A. Rotem , L. Etgar , J. Mater. Chem. A 2015, 3, 9171.

[89] T. Ma , M. Cagnoni , D. Tadaki , A. Hirano‐Iwata , M. Niwano , J. Mater. Chem. A 2015, 3, 14195.

[90] A. Dualeh , N. Tétreault , T. Moehl , P. Gao , M. K. Nazeeruddin , M. Grätzel , Adv. Funct. Mater. 2014, 24, 3250.

[91] M. Saliba , K. W. Tan , H. Sai , D. T. Moore , T. Scott , W. Zhang , L. A. Estroff , U. Wiesner , H. J. Snaith , J. Phys. Chem. C 2014, 118, 17171.

[92] C. Bi , Y. Shao , Y. Yuan , Z. Xiao , C. Wang , Y. Gao , J. Huang , J. Mater. Chem. A 2014, 2, 18508.

[93] M.‐F. Xu , H. Zhang , S. Zhang , H. L. Zhu , H.‐M. Su , J. Liu , K. S. Wong , L.‐S. Liao , W. C. H. Choy , J. Mater. Chem. A 2015, 3, 14424.

[94] R. Kang , J.‐E. Kim , J.‐S. Yeo , S. Lee , Y.‐J. Jeon , D.‐Y. Kim , J. Phys. Chem. C 2014, 118, 26513.

[95] W. Nie , H. Tsai , R. Asadpour , J.‐C. Blancon , A. J. Neukirch , G. Gupta , J. J. Crochet , M. Chhowalla , S. Tretiak , M. A. Alam , H.‐L. Wang , A. D. Mohite , Science 2015, 347, 522. 25635093 10.1126/science.aaa0472

[96] Y. C. Zheng , S. Yang , X. Chen , Y. Chen , Y. Hou , H. G. Yang , Chem. Mater. 2015, 27, 5116.

[97] Z. Xiao , Q. Dong , C. Bi , Y. Shao , Y. Yuan , J. Huang , Adv. Mater. 2014, 26, 6503. 25158905 10.1002/adma.201401685

[98] H. Yu , X. Liu , Y. Xia , Q. Dong , K. Zhang , Z. Wang , Y. Zhou , B. Song , Y. Li , J. Mater. Chem. A 2016, 4, 321.

[99] J. Liu , C. Cao , X. He , Q. Ye , L. Ouyang , D. Zhuang , C. Liao , J. Wei , W. Lau , ACS Appl. Mater. Interfaces 2015, 7, 24008. 26485481 10.1021/acsami.5b06780

[100] D. Liu , L. Wu , C. Li , S. Ren , J. Zhang , W. Li , L. Feng , ACS Appl. Mater. Interfaces 2015, 7, 16330. 26154760 10.1021/acsami.5b03324

[101] Z. Ren , A. Ng , Q. Shen , H. C. Gokkaya , J. Wang , L. Yang , W.‐K. Yiu , G. Bai , A. B. Djurisic , W. W.‐f. Leung , J. Hao , W. K. Chan , C. Surya , Sci. Rep. 2014, 4, 6752. 25341527 10.1038/srep06752 PMC4208060

[102] A. Ng , Z. Ren , Q. Shen , S. H. Cheung , H. C. Gokkaya , G. Bai , J. Wang , L. Yang , S. K. So , A. B. Djurisic , W. W.‐f. Leung , J. Hao , W. K. Chanf , C. Surya , J. Mater. Chem. A 2015, 3, 9223.

[103] J. You , Y. M. Yang , Z. Hong , T.‐B. Song , L. Meng , Y. Liu , C. Jiang , H. Zhou , W.‐H. Chang , G. Li , Y. Yang , Appl. Phys. Lett. 2014, 105, 183902.

[104] H. Gao , C. Bao , F. Li , T. Yu , J. Yang , W. Zhu , X. Zhou , G. Fu , Z. Zou , ACS Appl. Mater. Interfaces 2015, 7, 9110. 25871284 10.1021/acsami.5b00895

[105] A. M. A. Leguy , Y. Hu , M. Campoy‐Quiles , M. I. Alonso , O. J. Weber , P. Azarhoosh , M. v. Schilfgaarde , M. T. Weller , T. Bein , J. Nelson , P. Docampo , P. R. F. Barnes , Chem. Mater. 2015, 27, 3397.

[106] J. F. Galisteo‐López , M. Anaya , M. E. Calvo , H. Míguez , J. Phys. Chem. Lett. 2015, 6, 2200. 26266592 10.1021/acs.jpclett.5b00785 PMC4603615

[107] S. R. Raga , M.‐C. Jung , M. V. Lee , M. R. Leyden , Y. Kato , Y. Qi , Chem. Mater. 2015, 27, 1597.

[108] B. Yang , O. Dyck , J. Poplawsky , J. Keum , S. Das , A. Puretzky , T. Aytug , P. C. Joshi , C. M. Rouleau , G. Guscher , D. B. Geohegan , K. Xiao , Angew. Chem. Int. Ed. 2015, 54, 14862. 10.1002/anie.201505882 26486584

[109] S. Pathak , A. Sepe , A. Sadhanala , F. Deschler , A. Haghighirad , N. Sakai , K. C. Goedel , S. D. Stranks , N. Noel , M. Price , S. Hüttner , N. A. Hawkins , R. H. Friend , U. Steiner , H. J. Snaith , ACS Nano 2015, 9, 2311. 25712705 10.1021/nn506465n

[110] Y. Rong , Z. Tang , Y. Zhao , X. Zhong , S. Venkatesan , H. Graham , M. Patton , Y. Jing , A. M. Guloy , Y. Yao , Nanoscale 2015, 7, 10595. 26037081 10.1039/c5nr02866c

[111] H. Chen , Z. Wei , H. He , X. Zheng , K. S. Wong , S. Yang , Adv. Energy Mater. 2016, 6, 1502087.

[112] N. J. Jeon , J. H. Noh , Y. C. Kim , W. S. Yang , S. Ryu , S. I. Seok , Nat. Mater. 2014, 13, 897. 24997740 10.1038/nmat4014

[113] M. Xiao , F. Huang , W. Huang , Y. Dkhissi , Y. Zhu , J. Etheridge , A. Gray‐Weale , U. Bach , Y.‐B. Cheng , L. Spiccia , Angew. Chem. Int. Ed. 2014, 53, 9898. 10.1002/anie.201405334 25047967

[114] Y. Zhou , M. Yang , W. Wu , A. L. Vasiliev , K. Zhu , N. P. Padture , J. Mater. Chem. A 2015, 3, 8178.

[115] D. Shi , V. Adinolfi , R. Comin , M. Yuan , E. Alarousu , A. Buin , Y. Chen , S. Hoogland , A. Rothenberger , K. Katsiev , Y. Losovyj , X. Zhang , P. A. Dowben , O. F. Mohammed , E. H. Sargent , O. M. Bakr , Science 2015, 347, 519. 25635092 10.1126/science.aaa2725

[116] Q. Dong , Y. Fang , Y. Shao , P. Mulligan , J. Qiu , L. Cao , J. Huang , Science 2015, 347, 967. 25636799 10.1126/science.aaa5760

[117] Y. Dang , Y. Liu , Y. Sun , D. Yuan , X. Liu , W. Lu , G. Liu , H. Xia , X. Tao , CrystEngComm 2015, 17, 665.

[118] M. I. Saidaminov , A. L. Abdelhady , B. Murali , E. Alarousu , V. M. Burlakov , W. Peng , I. Dursun , L. Wang , Y. He , G. Maculan , A. Goriely , T. Wu , O. F. Mohammed , O. M. Bakr , Nat. Commun. 2015, 6, 7586. 26145157 10.1038/ncomms8586 PMC4544059

[119] G. Maculan , A. D. Sheikh , A. L. Abdelhady , M. I. Saidaminov , M. A. Haque , B. Murali , E. Alarousu , O. F. Mohammed , T. Wu , O. M. Bakr , J. Phys. Chem. Lett. 2015, 6, 3781. 26722870 10.1021/acs.jpclett.5b01666

[120] Y. Liu , Z. Yang , D. Cui , X. Ren , J. Sun , X. Liu , J. Zhang , Q. Wei , H. Fan , F. Yu , X. Zhang , C. Zhao , S. F. Liu , Adv. Mater. 2015, 27, 5176. 26247401 10.1002/adma.201502597

[121] Y. Fang , Q. Dong , Y. Shao , Y. Yuan , J. Huang , Nat. Photonics 2015, 9, 679.

[122] T. Zhang , M. Yang , E. E. Benson , Z. Li , J. v. d. Lagemaat , J. M. Luther , Y. Yan , K. Zhu , Y. Zhao , Chem. Commun. 2015, 51, 7820. 10.1039/c5cc01835h 25853846

[123] P. Zhao , J. Xu , X. Dong , L. Wang , W. Ren , L. Bian , A. Chang , J. Phys. Chem. Lett. 2015, 6, 2622. 26266744 10.1021/acs.jpclett.5b01017

[124] M. I. Saidaminov , A. L. Abdelhady , G. Maculana , O. M. Bakr , Chem. Commun. 2015, 51, 17658. 10.1039/c5cc06916e 26511771

[125] Q. Han , S.‐H. Bae , P. Sun , Y.‐T. Hsieh , Y. M. Yang , Y. S. Rim , H. Zhao , Q. Chen , W. Shi , G. Li , Y. Yang , Adv. Mater. 2016, 10.1002/adma.201505002. 10.1002/adma.201505002 26790006

[126] H. ‐H. Fang , R. Raissa , M. Abdu‐Aguye , S. Adjokatse , G. R. Blake , J. Even , M. A. Loi , Adv. Funct. Mater. 2015, 25, 2378.

[127] G. Walters , B. R. Sutherland , S. Hoogland , D. Shi , R. Comin , D. P. Sellan , O. M. Bakr , E. H. Sargent , ACS Nano 2015, 9, 9340. 26196162 10.1021/acsnano.5b03308

[128] T. Yamada , Y. Yamada , H. Nishimura , Y. Nakaike , A. Wakamiya , Y. Murata , Y. Kanemitsu , Adv. Electron. Mater. 2016, 2, 1500290.

[129] J. ‐S. Park , S. Choi , Y. Yan , Y. Yang , J. M. Luther , P. Parilla , K. Zhu , J. Phys. Chem. Lett. 2015, 6, 4304. 26722966 10.1021/acs.jpclett.5b01699

[130] Y. Yang , Y. Yan , M. Yang , S. Choi , K. Zhu , J. M. Luther , M. C. Beard , Nat. Commun. 2015, 6, 7961. 26245855 10.1038/ncomms8961 PMC4918347

[131] D. A. Valverde‐Chávez , C. S. Ponseca Jr ., C. C. Stoumpos , A. Yartsev , M. G. Kanatzidis , V. Sundström , D. G. Cooke , Energy Environ. Sci. 2015, 8, 3700.

[132] T. Baikie , N. S. Barrow , Y. Fang , P. J. Keenan , P. R. Slater , R. O. Piltz , M. Gutmann , S. G. Mhaisalkar , T. J. White , J. Mater. Chem. A 2015, 3, 9298.

[133] G. Grancini , V. D'Innocenzo , E. R. Dohner , N. Martino , A. R. S. Kandada , E. Mosconi , F. D. Angelis , H. I. Karunadasa , E. T. Hokee , A. Petrozza , Chem. Sci. 2015, 6, 7305. 10.1039/c5sc02542g PMC5512535 28757989

[134] Y. Bekenstein , B. A. Koscher , S. W. Eaton , P. Yang , A. P. Alivisatos , J. Am. Chem. Soc. 2015, 137, 16008. 26669631 10.1021/jacs.5b11199

[135] Y. Lin , Z. Yuan , Y. Tian , X. Wang , J. C. Wang , Y. Xin , K. Hanson , B. Ma , H. Gao , Adv. Mater. 2016, 28, 305. 26572239 10.1002/adma.201503954

[136] Z. Yuan , Y. Shu , Y. Tian , Y. Xin , B. Ma , Chem. Commun 2015, 51, 16385. 10.1039/c5cc06750b 26411807

[137] W. Tian , C. Zhao , J. Leng , R. Cui , S. Jin , J. Am. Chem. Soc. 2015, 137, 12458. 26390276 10.1021/jacs.5b08045

[138] J. A. Sichert , Y. Tong , N. Mutz , M. Vollmer , S. Fischer , K. Z. Milowska , R. G. Cortadella , B. Nickel , C. Cardenas‐Daw , J. K. Stolarczyk , A. S. Urban , J. Feldmann , Nano Lett. 2015, 15, 6521. 26327242 10.1021/acs.nanolett.5b02985

[139] S. T. Ha , X. Liu , Q. Zhang , D. Giovanni , T. C. Sum , Q. Xiong , Adv. Optical Mater. 2014, 2, 838.

[140] Q. Zhang , S. T. Ha , X. Liu , T. C. Sum , Q. Xiong , Nano Lett. 2014, 14, 5995. 25118830 10.1021/nl503057g

[141] Q. Liao , K. Hu , H. Zhang , X. Wang , J. Yao , H. Fu , Adv. Mater. 2015, 27, 3405. 25903387 10.1002/adma.201500449

[142] P. Tyagi , S. M. Arveson , W. A. Tisdale , J. Phys. Chem. Lett. 2015, 6, 1911. 26263268 10.1021/acs.jpclett.5b00664

[143] Q. A. Akkerman , S. G. Motti , A. R. S. Kandada , E. Mosconi , V. D'Innocenzo , G. Bertoni , S. Marras , B. A. Kamino , L. Miranda , F. D. Angelis , A. Petrozza , M. Prato , L. Manna , J. Am. Chem. Soc. 2016, 138, 1010. 26726764 10.1021/jacs.5b12124 PMC4731826

[144] D. M. Jang , K. Park , D. H. Kim , J. Park , F. Shojaei , H. S. Kang , J.‐P. Ahn , J. W. Lee , J. K. Song , Nano Lett. 2015, 15, 5191. 26161637 10.1021/acs.nanolett.5b01430

[145] L. Dou , A. B. Wong , Y. Yu , M. Lai , N. Kornienko , S. W. Eaton , A. Fu , C. G. Bischak , J. Ma , T. Ding , N. S. Ginsberg , L.‐W. Wang , A. P. Alivisatos , P. Yang , Science 2015, 349, 1518. 26404831 10.1126/science.aac7660

[146] R. Naphade , S. Nagane , G. S. Shanker , R. Fernandes , D. Kothari , Y. Zhou , N. P. Padture , S. Ogale , ACS Appl. Mater. Interfaces 2016, 8, 854. 26690942 10.1021/acsami.5b10208

[147] Y. Fu , F. Meng , M. B. Rowley , B. J. Thompson , M. J. Shearer , D. Ma , R. J. Hamers , J. C. Wright , S. Jin , J. Am. Chem. Soc. 2015, 137, 5810. 25871732 10.1021/jacs.5b02651

[148] E. Horváth , M. Spina , Z. Szekrényes , K. Kamarás , R. Gaal , D. Gachet , L. Forró , Nano Lett. 2014, 14, 6761. 25354371 10.1021/nl5020684

[149] F. Zhu , L. Men , Y. Guo , Q. Zhu , U. Bhattacharjee , P. M. Goodwin , J. W. Petrich , E. A. Smith , J. Vela , ACS Nano 2015, 9, 2948. 25661423 10.1021/nn507020s

[150] J.‐H. Im , J. Luo , M. Franckevičius , N. Pellet , P. Gao , T. Moehl , S. M. Zakeeruddin , M. K. Nazeeruddin , M. Grätzel , N.‐G. Park , Nano Lett. 2015, 15, 2120. 25710268 10.1021/acs.nanolett.5b00046

[151] S. Zhuo , J. Zhang , Y. Shi , Y. Huang , B. Zhang , Angew. Chem. Int. Ed. 2015, 54, 5693. 10.1002/anie.201411956 25776103

[152] M. Spina , M. Lehmann , B. Náfrádi , L. Bernard , E. Bonvin , R. Gaál , A. Magrez , L. Forró , E. Horváth , Small 2015, 37, 4824. 10.1002/smll.201501257 26172855

[153] A. Fu , P. Yang , Nat. Mater. 2015, 14, 557. 25990907 10.1038/nmat4291

[154] J. Xing , X. F. Liu , Q. Zhang , S. T. Ha , Y. W. Yuan , C. Shen , T. C. Sum , Q. Xiong , Nano Lett. 2015, 15, 4571. 26043362 10.1021/acs.nanolett.5b01166

[155] A. B. Wong , M. Lai , S. W. Eaton , Y. Yu , E. Lin , L. Dou , A. Fu , P. Yang , Nano Lett. 2015, 15, 5519. 26192740 10.1021/acs.nanolett.5b02082

[156] J.‐H. Im , C.‐R. Lee , J.‐W. Lee , S.‐W. Park , N.‐G. Park , Nanoscale 2011, 3, 4088. 21897986 10.1039/c1nr10867k

[157] L. C. Schmidt , A. Pertegás , S. González‐Carrero , O. Malinkiewicz , S. Agouram , G. M. Espallargas , H. J. Bolink , R. E. Galian , J. Pérez‐Prieto , J. Am. Chem. Soc. 2014, 136, 850. 24387158 10.1021/ja4109209

[158] S. Gonzalez‐Carrero , R. E. Galian , J. Pérez‐Prieto , J. Mater. Chem. A 2015, 3, 9187.

[159] F. Zhang , H. Zhong , C. Chen , X.‐g. Wu , X. Hu , H. Huang , J. Han , B. Zou , Y. Dong , ACS Nano 2015, 9, 4533. 25824283 10.1021/acsnano.5b01154

[160] H. Huang , A. S. Susha , S. V. Kershaw , T. F. Hung , A. L. Rogach , Adv. Sci. 2015, 2, 1500194. 10.1002/advs.201500194 PMC5115379 27980980

[161] L. Protesescu , S. Yakunin , M. I. Bodnarchuk , F. Krieg , R. Caputo , C. H. Hendon , R. X. Yang , A. Walsh , M. V. Kovalenko , Nano Lett. 2015, 15, 3692. 25633588 10.1021/nl5048779 PMC4462997

[162] J. Pan , S. P. Sarmah , B. Murali , I. Dursun , W. Peng , M. R. Parida , J. Liu , L. Sinatra , N. Alyami , C. Zhao , E. Alarousu , T. K. Ng , B. S. Ooi , O. M. Bakr , O. F. Mohammed , J. Phys. Chem. Lett. 2015, 6, 5027. 26624490 10.1021/acs.jpclett.5b02460

[163] Y. Wang , X. Li , J. Song , L. Xiao , H. Zeng , H. Sun , Adv. Mater. 2015, 27, 7101. 26448638 10.1002/adma.201503573

[164] Y.‐S. Park , S. Guo , N. S. Makarov , V. I. Klimov , ACS Nano 2015, 9, 10386. 26312994 10.1021/acsnano.5b04584

[165] D. N. Dirin , S. Dreyfuss , M. I. Bodnarchuk , G. Nedelcu , P. Papagiorgis , G. Itskos , M. V. Kovalenko , J. Am. Chem. Soc. 2014, 136, 6550. 24746226 10.1021/ja5006288 PMC4524702

[166] Z. Ning , X. Gong , R. Comin , G. Walters , F. Fan , O. Voznyy , E. Yassitepe , A. Buin , S. Hoogland , E. H. Sargent , Nature 2015, 523, 324. 26178963 10.1038/nature14563

[167] J. Peng , Y. Chen , X. Zhang , A. Dong , Z. Liang , Adv. Sci., 2016, 3,1500432. 10.1002/advs.201500432 PMC5067684 27812473

[168] I. V. Markov , Crystal Growth for Beginners, World Scientific, Singapore, 1995.

[169] S. Yang , Y. C. Zheng , Y. Hou , X. Chen , Y. Chen , Y. Wang , H. Zhao , H. G. Yang , Chem. Mater. 2014, 26, 6705.

[170] O. Horváth , I. Mikó , J. Photochem. Photobiol. A 1998, 114, 95.

[171] Y. Chen , S. Yang , X. Chen , Y. C. Zheng , Y. Hou , Y. H. Li , H. D. Zeng , H. G. Yang , J. Mater. Chem. A 2015, 3, 15854.

[172] M. I. Saidaminov , V. Adinolfi , R. Comin , A. L. Abdelhady , W. Peng , I. Dursun , M. Yuan , S. Hoogland , E. H. Sargent , O. M. Bakr , Nat. Commun. 2015, 6, 8724. 26548941 10.1038/ncomms9724 PMC4667636

